# Fasudil increases temozolomide sensitivity and suppresses temozolomide-resistant glioma growth via inhibiting ROCK2/ABCG2

**DOI:** 10.1038/s41419-017-0251-9

**Published:** 2018-02-07

**Authors:** Xin Zhang, Xiuting Liu, Wei Zhou, Mengdi Yang, Yang Ding, Qing Wang, Rong Hu

**Affiliations:** 1 State Key Laboratory of Natural Medicines, Department of Physiology, China Pharmaceutical University, Nanjing, Jiangsu 210009 China; 2 Department of Neurosurgery, Wuxi Second Hospital Affiliated Nanjing Medical University, Wuxi, Jiangsu 214002 China

## Abstract

Resistance to temozolomide (TMZ) is a major clinical challenge in glioma treatment, but the mechanisms of TMZ resistance are poorly understood. Here, we provided evidence that ROCK2 acted redundantly to maintain resistance of TMZ in TMZ-resistant gliomas, and as a ROCK2 phosphorylation inhibitor, fasudil significantly suppressed proliferation of TMZ-resistant gliomas in vivo and vitro via enhancing the chemosensitivity of TMZ. Additionally, the membrane translocation of ABCG2 was decreased with fasudil by ROCK2/moesin pathway. We also showed that fasudil suppressed the expression of ABCG2 via ROCK2/moesin/β-catenin pathway. Our results reveal an indispensable role for ROCK2 and provide strong evidence for the therapeutic use of fasudil in the clinical setting for TMZ-resistant gliomas.

## Introduction

The emergence of tumor cell resistance to chemotherapy represents a major challenge for the development of durable therapeutic strategies across most solid cancers including glioma, the most malignant primary brain tumor in adults. The current standard adjuvant therapeutic strategy for gliomas is maximum surgery followed by radiotherapy with the alkylating agent temozolomide (TMZ)^1^. Subgroup analyses indicate that the TMZ benefit is limited due to TMZ resistance^2^. O^6^-methylguanine-DNA methyltransferase (MGMT) was overactivated in the tissues of patients resistant to TMZ^3^. Moreover, other mechanisms contribute to TMZ resistance such as overexpression of murine double minute 2 (MDM2)^4^, p53 gene mutation^5,6^, epidermal growth factor receptor^5^ and galectin-1^7^. To increase the effectiveness of TMZ, different therapeutic molecules have been developed^8–10^. However, the systemically administered treatments lacked selectivity. Moreover, the drugs which target gliomas must cross the blood–brain barrier (BBB), which was difficult to achieve. For these reasons, TMZ-resistant gliomas remain incurable at present and new effective strategies to deliver innovative drugs at their targets are strongly needed.

A clinical agent, fasudil (HA-1077), was approved in 1995 for the treatment of cerebral vasospasm in China and Japan^11^. Fasudil was an inhibitor of Rho-associated protein kinases (ROCKs or Rho kinases), and was reported to increase cisplatin-induced growth inhibition and apoptosis in ovarian cancer cells through suppressing phosphorylation of ROCKs^12,13^. It also sensitized pancreatic cancer cells to chemotherapy^14^. Growing evidence suggests that ROCKs play an important role in mediating resistance to chemotherapeutics^11,15,16^. ROCKs regulate the cytoskeleton through the phosphorylation of myosin light chain (MLC) and myosin phosphatase. Recent studies indicate that ROCK inhibition can enhance cisplatin-induced cytotoxicity via suppressing hypoxia-inducible factor-1 signaling, which is downstream of the pathway of ROCKs^17–19^. ROCK inhibitors Y-27632 and fasudil increased the sensibility of gemcitabine in pancreatic cancer stem cells^20^. In malignant glioma, ROCK1 knockdown increased the efficacy of nimustine hydrochloride^21^. Downregulation of ROCK2 through nanocomplex sensitized the cytotoxic effect of temozolomide in U251 cells^22^. Thus, we hypothesized that ROCKs may play an important role, but the potential mechanisms of ROCK inhibition in chemoresistance of gliomas remain unclear. Here, we tested the efficacy of fasudil against TMZ-resistant gliomas to investigate the potential roles of ROCKs in TMZ-resistant gliomas.

One of ROCK downstream effectors, ezrin-radixin-moesin (ERM) proteins played a critical role in drug resistance in the MOLT4 cell line^23^. ERM proteins were involved in the excretion of cisplatin in the small intestine^24^, and regulated the insertion of P-glycoprotein (P-gP) on the intercellular membrane in multidrug-resistant breast cancer cells^25^. One member of the ERM family of proteins, moesin, was reported overexpressed in glioblastoma (GBM), but two other ERM proteins, ezrin and radixin, show no significant differential expression, and knockdown of moesin alone reduced the migration of GBM cells^26^. ATP-binding cassette (ABC) transporter is reported involved in regulation efficacy of chemo-agents in brain tumors. With TMZ treatment, intracellular P-gP was trafficked to the cell membrane and conformational change into active P-gP. At the later phase, gene transcription of P-gP was induced by TMZ^27^. Concomitant inhibition of P-gP and ATP-binding cassette sub-family G member 2 (ABCG2) by elacridar may further improve the efficacy of ABT-888+TMZ combination treatment in  GBM^28^. The multidrug resistance protein 1 (MRP1) inhibition led to a significant increase in vincristine- and etoposide-induced cell death in cells derived from recurrent grade IV GBM^29^. Taken together, we speculate that ROCKs/moesin/ABC transporter may play a role in resistance of TMZ.

In this study, we demonstrated that glioma TMZ resistance was reversed by addition of TMZ combined with fasudil. This mechanism may involve the inhibition of the ROCKs/moesin/ABC transporter.

## Results

### ROCK2 was upregulated in TMZ-resistant cells and fasudil increased sensibility of TMZ to overcome resistance

As shown in supplementary table S1, the half-maximal inhibitory concentration (IC50) values of adapted cells were much higher than parental cells (U87, U251, T5 and T6), indicating that the TMZ-resistant (TMZ-R) cell lines (U251R, U87R, T5R and T6R) were successfully established. rG-1 was a primary glioma cell which was resistant to TMZ (supplementary table S1A). Supplementary table S1B shows that TMZ-R cells generated more colonies than corresponding glioma cells under stimulation of different concentrations of TMZ for 7 days, further demonstrating that the TMZ-R cell lines were successfully acquired in our study. It has been suggested that MGMT is the most important determinant of resistance to TMZ. However, we found that mgmt gene expression of U251R was upregulated, and no substantial changes were found in other TMZ-R cells (supplementary table S1C). More remarkably, MGMT protein expression in rG-1 cell was lower than U251R (supplementary table S1D).

**Fig. 1:**
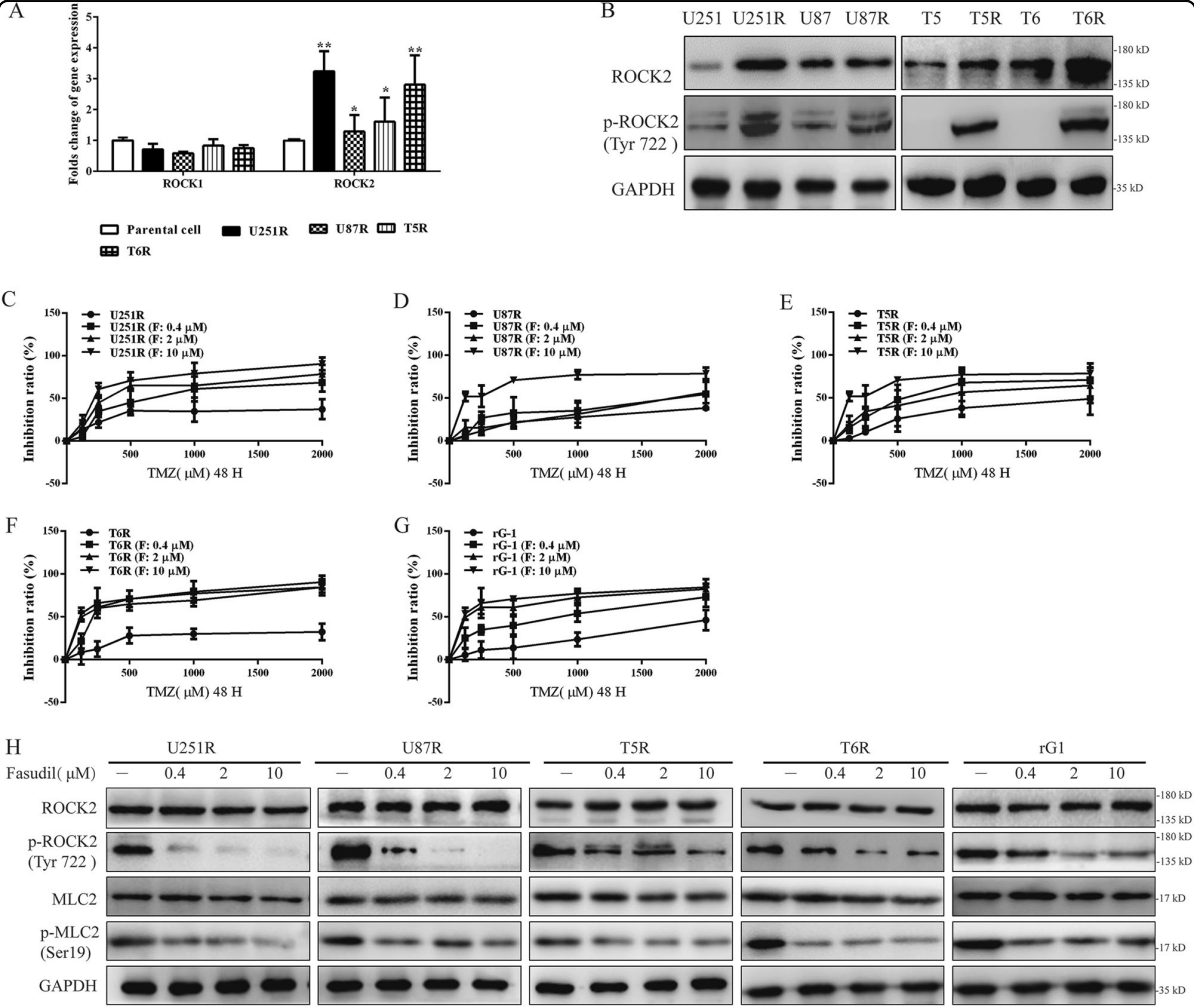
ROCK2 was upregulated in TMZ-R cells and fasudil increased sensibility of TMZ to overcome resistance. **a** Gene expression of ROCK1 and ROCK2 was determined in all cells. The expression levels of parental cells were set to 100%. **b** Protein levels of ROCK2 were detected by western blot. **c**–**g** TMZ-R cells (**c**: U251R, **d**: U87R, **e**: T5R, **f**: T6R, **g**: rG-1) were treated with different concentrations of fasudil (0.4, 2, 10 μM) and TMZ (0, 125, 250, 500, 1000 and 2000 μM), and then cell viability was determined by the MTT assay. **h** Expression of ROCK2, p-ROCK2, MLC2 and p-MLC2 was determined under stimulation of fasudil (0.4, 2, 10 μM). Statistical differences compared with the control group is given as *P < 0.05, **P < 0.01

Recent studies revealed that resistance of cisplatin was induced by ROCK overexpression. To determine ROCK expression in TMZ-R glioma cells, mRNAs of rock1 and rock2 were detected, and rock2 was upregulated in all resistance cells (Fig. 1a). We further found that ROCK2 and p-ROCK2 (Tyr 722) expression was increased in TMZ-R cells (Fig. 1b). In addition, as the upstream of ROCK2, activation of RhoA was determined in TMZ-R cells. As shown in supplementary Figure S2, levels of RhoA guanosine triphosphate (GTP) were increased and RhoA guanosine diphosphate (GDP) amount was reduced, indicating that RhoA/ROCK2 was activated in TMZ-R glioma cells. It was well understood that ROCK inhibition led to growth suppression and apoptosis by fasudil^13^. However, growth of rG-1 cell was increased with the absence of ROCK2 proteins (Supplementary Figure S3A). Interestingly, with TMZ stimulation, knockdown (KD) of ROCK2 caused proliferation defects in TMZ-R cells (Supplementary Figure S3A and S3B). These results revealed that ROCK2 KD increased TMZ sensibility. Thus, we speculated that TMZ sensibility may increase by ROCK2 inhibitor fasudil.

To investigate the effect of fasudil on TMZ-R cells, TMZ-R cells were treated with fasudil (0.2, 4, 10 μM) and TMZ (0, 125, 250, 500, 1000, 2000 μM) for 48 h. It was found that fasudil increased sensibility of TMZ to inhibit TMZ-R cell proliferation (Fig. 1c, g), and IC50 of cells was decreased under stimulation of fasudil (Table 1). We also found that expression of p-ROCK2 was reduced in all TMZ-R cells in a dose-dependent manner (Fig. 1h). As a substrate of ROCKs, p-MLC2 level was reduced by fasudil, suggesting that ROCK2 was inhibited (Fig. 1h). However, fasudil, as a selective inhibitor of ROCKs, did not affect RhoA GTP and GDP expressions in TMZ-R cells (Supplementary Figure S2).

**Table 1:** The IC50 (TMZ: μM) of TMZ under stimulation of fasudil (0.4, 2, 10 μM)

	IC50 (TMZ: μM)			
	–	F: 0.4 μM	F: 2 μM	F: 10 μM
U251R	4189.00 ± 922.61	853.66 ± 210.39^*^	440.26 ± 55.41^**^	244.7 ± 56.01^**^
U87R	3262.00 ± 1079.21	1326.66 ± 310.68^*^	1566.66 ± 269.78^**^	176.16 ± 40.08^*^
T5R	1731.33 ± 363.08	705.16 ± 107.80^*^	574.20 ± 172.59^**^	150.50 ± 44.17^*^
T6R	5178.66 ± 957.04	407.63 ± 150.78^*^	198.76 ± 69.30^*^	101.93 ± 14.55^*^
rG-1	2868.66 ± 350.48	852.36 ± 214.80^*^	213.36 ± 161.17^**^	86.53 ± 32.96^*^
U87 neurospheres	3629.66 ± 354.45	2143.66 ± 905.64	1128.66 ± 123.71^*^	236.43 ± 47.08^**^

Statistical differences compared with the control group (U251/U87/T5/T6/C6) is given as *P < 0.05, **P < 0.01

We confirmed that ROCK2 played an important role for maintaining TMZ resistant in both MGMT positive and negative TMZ-R cell. We also showed that depletion of ROCK2 by fasudil increased sensibility of TMZ.

### Fasudil regulated total and membrane expression of ABCG2

**Fig. 2:**
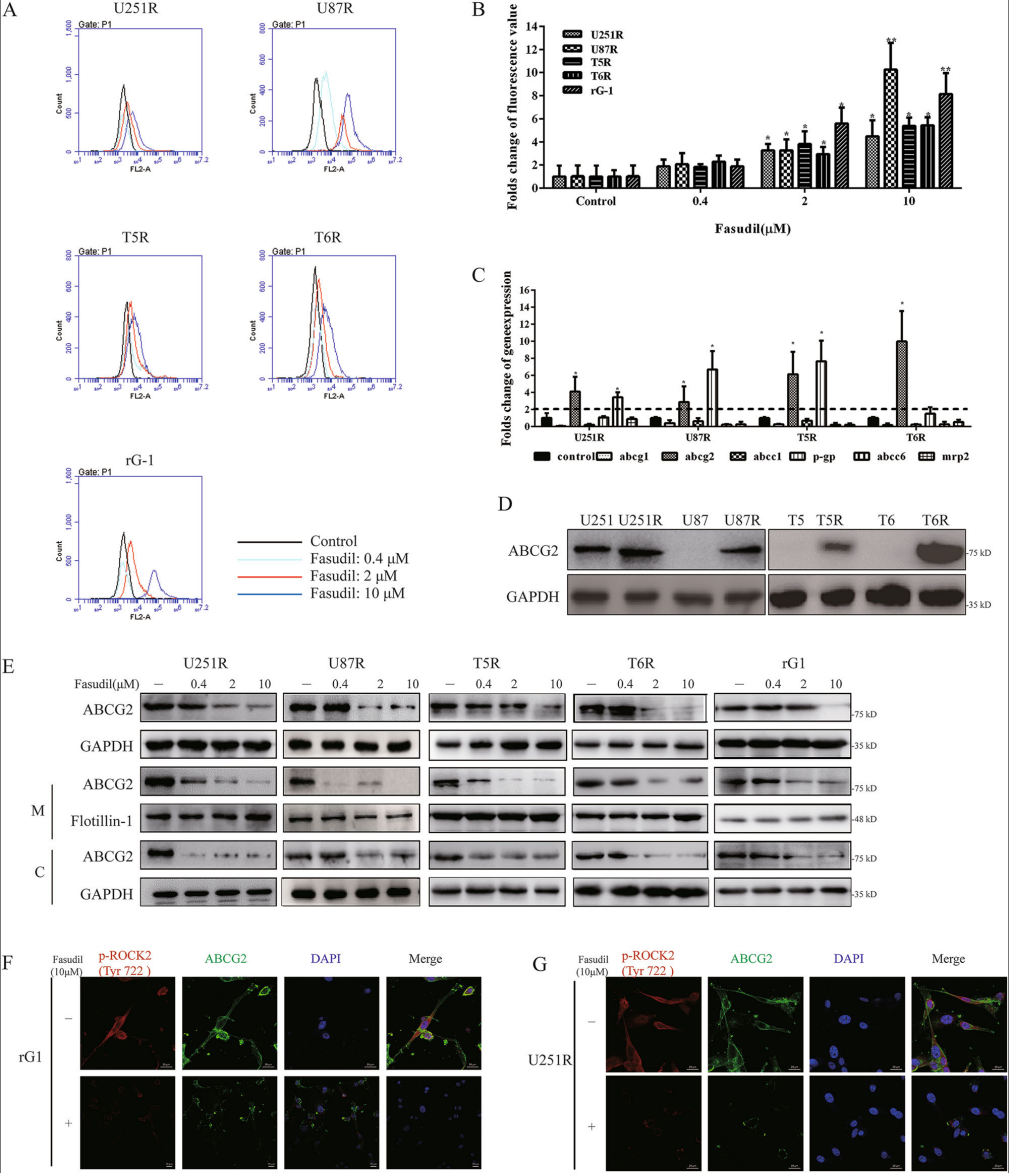
Fasudil regulated total and membrane expression of ABCG2. **a**, **b** Cells were treated with fasudil (0.4, 2, 10 μM), and accumulation of DOX were tested. **c** mRNA expression of abcg1, abcg2, p-gp, abcc1, abcc6 and mrp2 was detected by real-time PCR. The expression levels of parental cells were set to 100%. **d** Protein levels of ABCG2 were determined in resistance and parental cells. **e** All resistant cells were treated with fasudil (0.4, 2, 10 μM) for 24 h, and total, membrane (M) and cytoplasm (c) expression of ABCG2 was examined by western blot. **f**, **g** U251R (**g**) and rG-1 (**f**) were stimulated with fasudil (10 μM), IF was used to analyze expression of p-ROCK2 and ABCG2 (p-ROCK2: red, ABCG2: green; scale bar, 20 μm). GAPDH and flotillin-1 were used as loading control. Statistical differences compared with the control group is given as *P < 0.05, **P < 0.01

After stimulation with RhoGTPase, ROCK2 translocates from the cytosol to the membrane^16^ and regulates the translocation of ABC transporters to the membrane^30^. Inhibition of ROCKs led to accumulation of cisplatin in intestinal epithelial cells^25^. Therefore, we hypothesized that via inhibiting ROCK2, fasudil may result in accumulation of TMZ in TMZ-R cells. To determine this speculation, doxorubicin (DOX) was used to test accumulation in TMZ-R cells with pretreatment of fasudil for 24 h. As shown in Fig. 2a, b, fasudil (2, 10 μM) remarkably increased accumulation of DOX. To investigate the potential mechanism, the expressions of ABC transporter genes including abcg1, abcg2, p-gp, abcc1, abcc6 and mrp2 were detected^31–35^. The gene of abcg2 was consistently highly expressed in TMZ-R cells, and p-gp was upregulated in U251R, U87R and T5R (Fig. 2c). We further confirmed that ABCG2 protein expression was increased in TMZ-R cells (Fig. 2d). Pharmacological inhibition of ABCG2 was reported to increase cell death following treatment with TMZ^36^. We used ABCG2 inhibitor Ko-143 (15 μM) to suppress function of ABCG2, and followed with TMZ. As shown in supplementary figure S4, proliferation was decreased in U251R and rG-1 cells. With knockdown of ABCG2 expression, cell growth was also suppressed with TMZ. With fasudil for 24 h, expression of ABCG2 in TMZ-R cells was downregulated in total, and protein levels of ABCG2 on cytomembrane (M) and cytoplasm (C) were decreased (Fig. 2e). Immunofluorescence (IF) results showed that ABCG2 was reduced in rG-1 and U251R cells with fasudil (Fig. 2f). These data demonstrated that fasudil regulated overall and membrane-specific expression of ABCG2 in TMZ resistance glioma cells.

### Expression of ABCG2 was downregulated by fasudil via inhibition of ROCK2/moesin/β-catenin

**Fig. 3:**
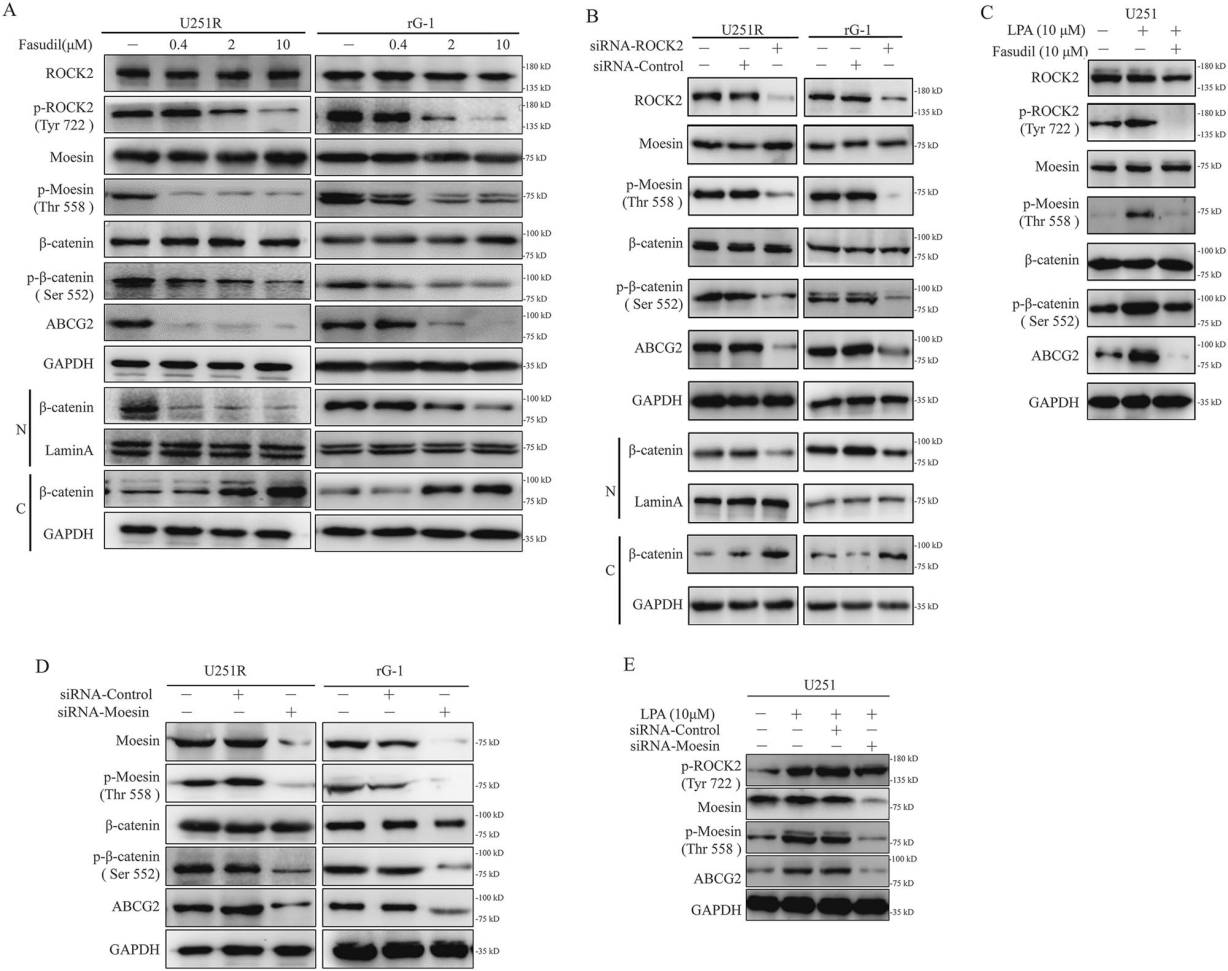
Expression of ABCG2 was downregulated by fasudil via inhibition of ROCK2/moesin/β-catenin. **a** U251R and rG-1 were stimulated by fasudil (0.4, 2, 10 μM) for 24 h, and protein levels of ROCK2, p-ROCK2, moesin, p-moesin (Thr 558), β-catenin, p-β-catenin (Ser 552) were detected by western blot. β-Catenin expressions of nucleus (N) and cytoplasm (C) were detected. **b** U251R and rG-1 cells were transfected with ROCK2-siRNA, p-moesin (Thr 558), p-β-catenin (Ser 552) and ABCG2 were detected, and nuclear accumulation of β-catenin was examined. **c** U251 cell was pretreated with LPA, and then cells were stimulated with fasudil, and the expression of ROCK2, p-ROCK2 (Tyr 722), moesin, p-moesin (Thr 558), β-catenin, p-β-catenin (Ser 552) and ABCG2 was determined by western blot.** d** U251R and rG-1 cells were transfected with moesin-siRNA, and protein levels of moesin, p-moesin (Thr 558), β-catenin, p-β-catenin (Ser 552) and ABCG2 were detected by western blot. **e** LPA was used to stimulate U251 with or without siRNA of moesin, and the expression of p-ROCK2 (Tyr 722), moesin and ABCG2 was examined by western blot. GAPDH and Lamin A were used as loading control

Moesin was downstream of ROCK2 and regarded as a glioma progression marker. Previous studies showed that β-catenin was downstream of moesin^37^. After stimulation of fasudil in U251R and rG-1 cells, p-moesin (Thr 558), p-β-catenin (Ser 552), ABCG2 expression and nuclear accumulation of β-catenin were decreased (Fig. 3a). The protein levels of ezrin and radixin were also similar in parental and TMZ-R cells. What is more, the phosphorylation of ezrin and radixin was also similar in these two cell lines (Supplementary Figure S5). Next, we found that expression of p-moesin, p-β-catenin, and ABCG2 was reduced with ROCK2 KD (Fig. 3b). It was reported that activation of ROCKs was mediated by lysophosphatidic acid (LPA). In U251 cells, p-ROCK2, p-moesin, p-β-catenin and ABCG2 protein levels were induced with LPA (10 μM). However, fasudil reversed this effect (Fig. 3c). Knockdown of moesin led to inhibition of p-β-catenin and ABCG2 in TMZ-R cells (Fig. 3d). As shown in Fig. 3e, U251 cells were pretreated with LPA, and knockdown of moesin resulted in absence of ABCG2. These results showed that fasudil reduced the expression of ABCG2 via moesin/β-catenin.

### Fasudil regulated ROCK2/moesin/ABCG2 on membrane of resistance cells

**Fig. 4:**
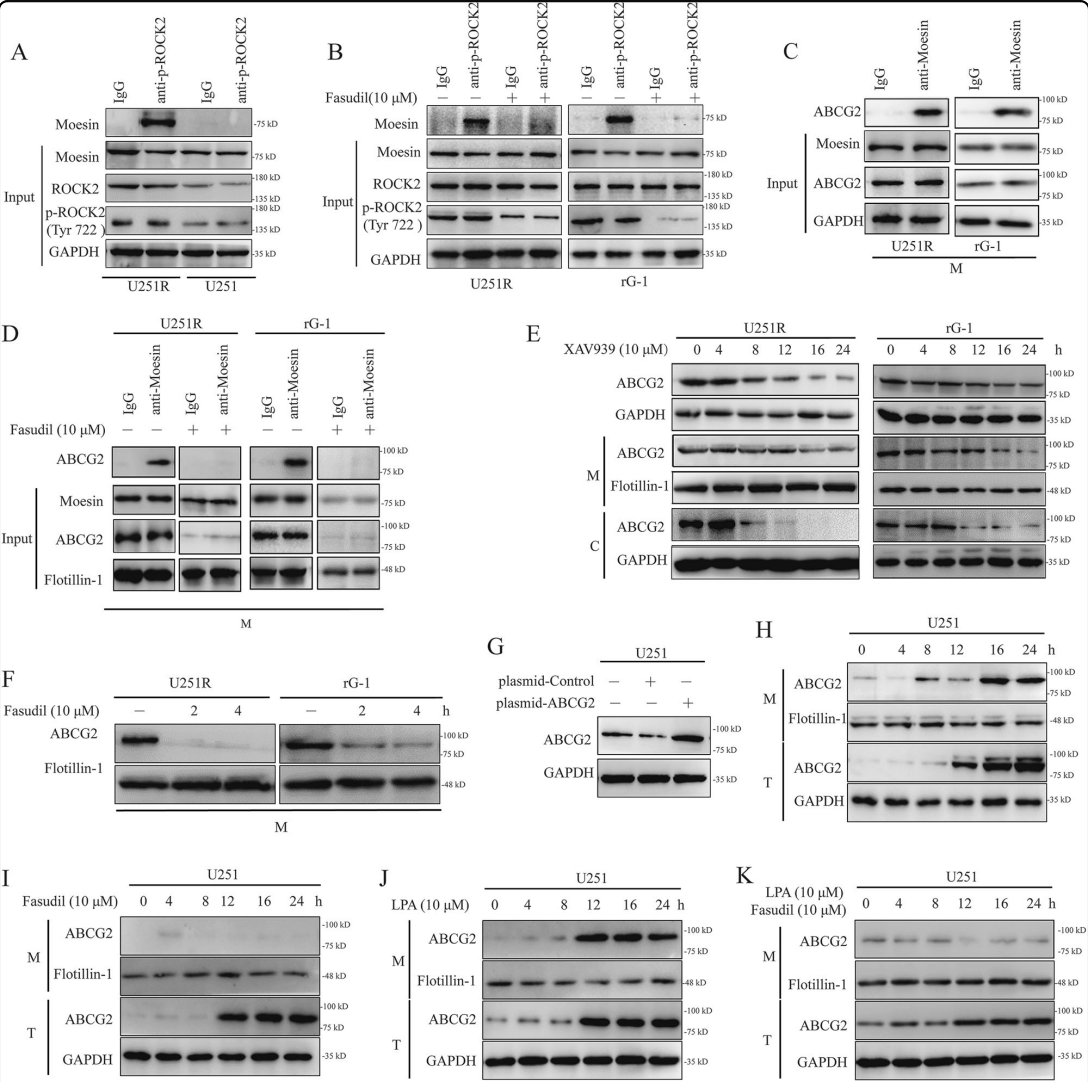
Fasudil regulated ROCK2/moesin/ABCG2 on membrane of TMZ-R cells. **a** IP studies were performed with p-ROCK2 and moesin using U251 and U251R. **b** U251R and rG-1 cells were treated with fasudil (10 μM) for 24 h, and IP was then performed to detect the interaction between p-ROCK2 and moesin. **c** The interaction of moesin and ABCG2 was detected by IP in U251R and rG-1 cells. **d** U251R and rG-1 cells were treated with fasudil (10 μM) for 24 h, and IP was then performed to detect the interaction between moesin and ABCG2. **e**, **f** U251R and rG-1 cells were treated with XAV939 (10 μM) for 8 h, and then cells were stimulated with fasudil for 2 and 4 h, and western blot was performed for ABCG2 expression. **g** U251 cell was transfected with ABCG2 overexpression plasmid, and ABCG2 protein was determined by western blot. **h** Transfected with ABCG2 plasmid, expression of ABCG2 of membrane (M) and total (T) was detected at different time points (0, 4, 8, 12, 16 and 24 h) in U251 cell. **i** U251 cell was transfected with ABCG2 overexpression plasmid, then fasudil (10 μM) was used to treat cell, and ABCG2 expression of membrane (M) and total (T) was determined at different time points (0, 4, 8, 12, 16, 24 h). **j** Transfected with ABCG2 plasmid, expression of ABCG2 of membrane (M) and total (T) was detected at different time points (0, 4, 8, 12, 16, 24 h) in U251 cell under treatment of LPA (10 μM). **k** U251 cell was transfected with ABCG2 overexpression plasmid, then cell was treated with LPA (10 μM) and fasudil (10 μM), ABCG2 expression of membrane (M) and total (T) was determined at different time points (0, 4, 8, 12, 16, 24 h). GAPDH and flotillin-1 were used as loading control

According to previous studies of ROCK/ERM/P-gP, we speculated that ROCK2 mediated moesin/ABCG2 on membrane, and fasudil might disrupt the interaction between ROCK2 and moesin, leading to decreased phosphorylation of moesin, and therefore blocking the translocation of ABCG2 to the membrane. To test this hypothesis, we found that ROCK2 and moesin were interconnected in U251R (Fig. 4a), and this interaction was disrupted by fasudil in U251R and rG-1 cells (Fig. 4b). Meanwhile, moesin and ABCG2 were shown to interact by immunoprecipitation (IP) assay (Fig. 4c). In U251R and rG-1 cells, fasudil obviously reduced interaction between moesin and ABCG2 (Fig. 4d). As a β-catenin inhibitor, XAV939 decreased the total expression of ABCG2 without decreasing membrane expression from 8 to 12 h (Fig. 4e). When U251R and rG-1 cell lines were treated with XAV939 for 8 h and then with fasudil for the next 4 h, the expression level of ABCG2 on the membrane was reduced (Fig. 4e, f). U251 cell was transfected with ABCG2 plasmid, and expression of ABCG2 was upregulated (Fig. 4g). After transfection, total expression of ABCG2 was upregulated at 12 h, whereas protein level of ABCG2 on membrane was increased at 16 h (Fig. 4h). To inhibit translocation of ABCG2, the ABCG2 plasmid transfection cells were treated with fasudil. As shown in Fig. 4i, on membrane, ABCG2 expression was suppressed. However, LPA promoted translocation of ABCG2 on membrane at 12 h, and total ABCG2 protein was upregulated and at the same time induced by plasmid. Next, with LPA pretreatment, fasudil led to a decrease of ABCG2 translocation on membrane in overexpression cell (Fig. 4k). These data confirmed that fasudil affected the membrane translocation of ABCG2 by ROCK2.

### Fasudil combined with TMZ inhibited TMZ-R glioma growth in vivo

**Fig. 5:**
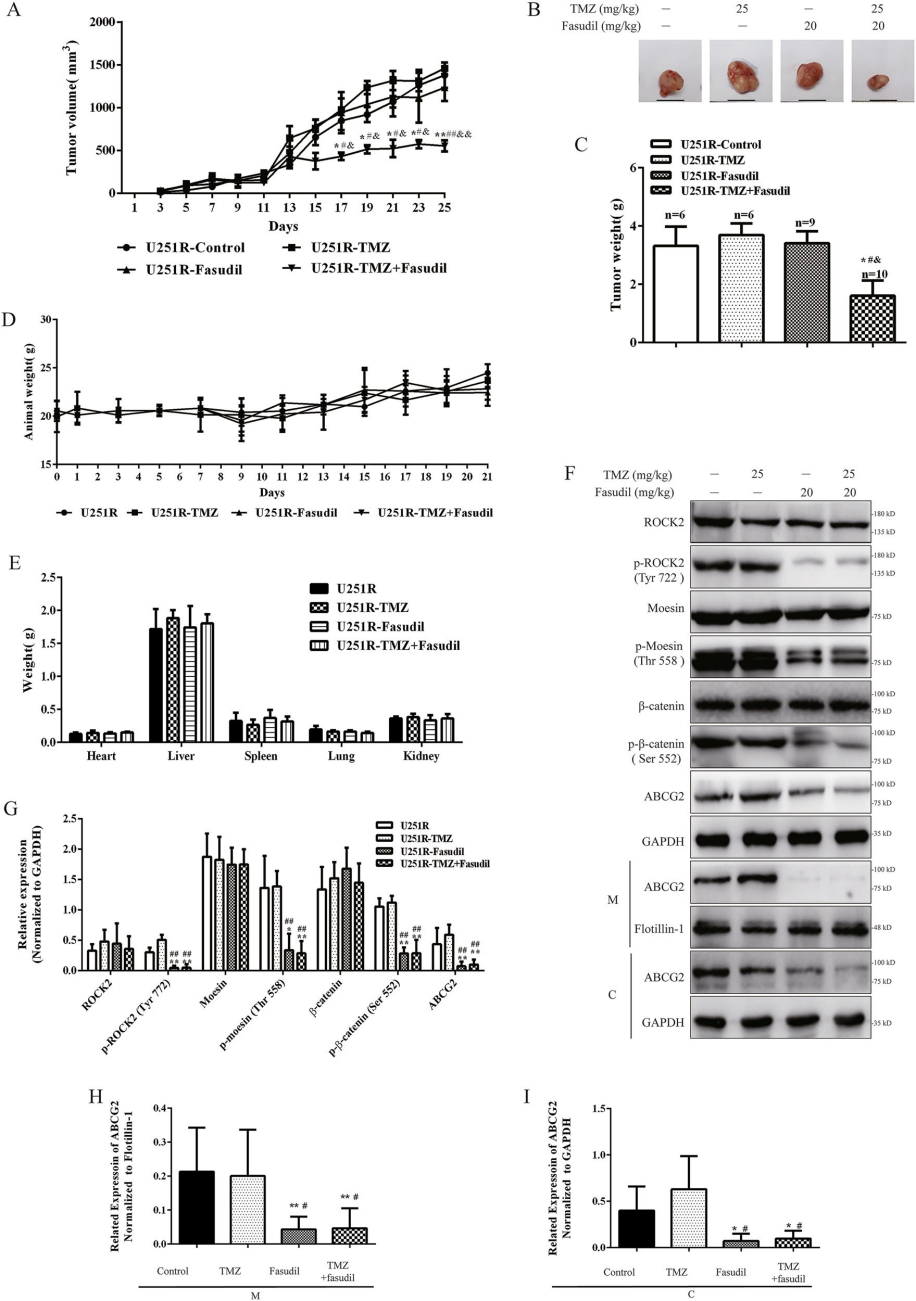
Fasudil combined with TMZ inhibited U251R cell growth in vivo. **a** TMZ resistance tumor size of nude mice was measured by vernier caliper for 21 days during the treatment time in all groups. **b**, **c** Weight of tumor obtained from tumor-bearing mice is shown. **d** Weight changes of nude mice in four separate groups were measured for 21 days. **e** Weight of heart, liver, spleen, lung, kidney and brain in four separate groups were measured in day 21. **f**, **g** ROCK2, p-ROCK2 (Tyr 722), moesin, p-moesin (Thr 558), β-catenin, p-β-catenin (Ser 552) and ABCG2 expression (total, membrane and cytoplasm) of tumors was determined by western blot and quantified (normalized to GAPDH). **h**, **i** The membrane and cytoplasm expressions of ABCG2 were quantified (normalized to flotillin-1 or GAPDH). GAPDH and flotillin-1 were used as loading control. Statistical differences compared with the control group is given as *P < 0.05, **P < 0.01, compared with the TMZ group is given as ^#^*P* < 0.05, ^##^*P* < 0.01, and compared with the fasudil group is given as ^&^*P* < 0.05, ^&&^*P* < 0.01

To determine the antitumor effects of fasudil in combination with TMZ in vivo, two types of animals were used to establish transplanted TMZ resistance glioma models. U251R cells were subcutaneously injected into nude mice to establish a xenograft tumor model. At day 17, the TMZ plus fasudil group showed significant inhibition of tumor growth (Fig. 5a). In comparison, the other groups exhibited no inhibitory effects throughout the entire treatment period (Fig. 5a). Tumor weight was measured after killing of mice at 21 days. As shown in Fig. 5b, c, a significant reduction in tumor weight was observed in the TMZ plus fasudil group compared with other groups, and there were no significant changes in body and organ weight among different groups (Fig. 5d, e), indicating a low toxicity of the combination treatment. The expression of p-ROCK2, p-moesin, p-β-catenin and ABCG2 was reduced in groups of fasudil and TMZ+fasudil (Fig. 5f, g). In the groups of fasudil and TMZ+fasudil, the amount of ABCG2 on membrane was also decreased (Fig. 5f, h, i).

**Fig. 6:**
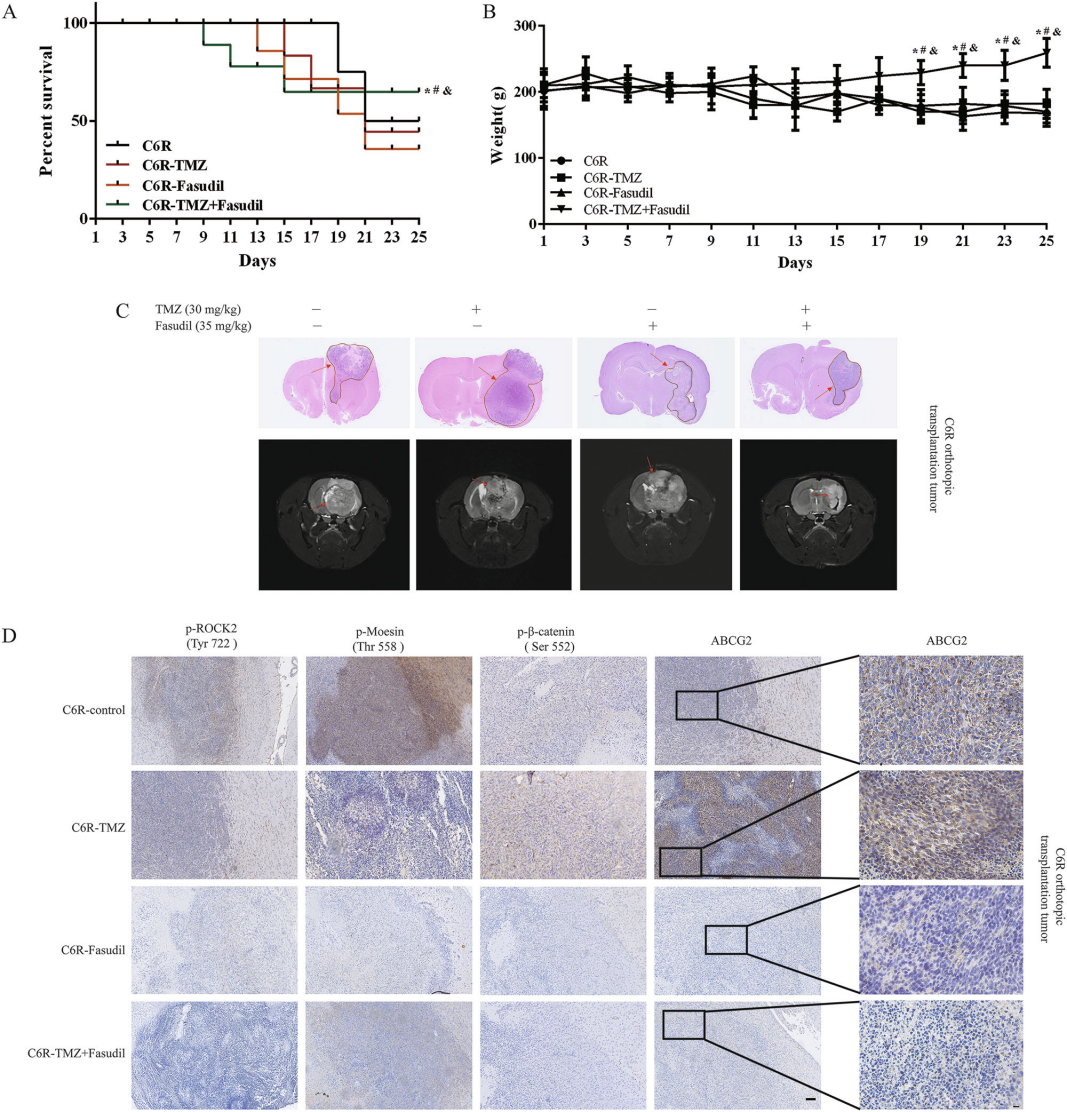
Fasudil combined with TMZ inhibited C6R cell growth in vivo. **a** The survival curve of glioma-bearing rats after various treatments. **b** Weight changes of TMZ-resistant glioma-bearing rats were measured for 25 days. **c** H&E-stained brain sections from TMZ-resistant glioma-bearing rats after 25 days (upper). Enhanced MRI was performed for TMZ-resistant glioma-bearing rats at day 24 (lower). **d** ROCK2, p-ROCK2 (Tyr 722), p-moesin (Thr 558), p-β-catenin (Ser 552) and ABCG2 expression of tumors was determined by IHC (scale bar, 50 μm). ABCG2 expression is also shown on the right column by IHC assay (scale bar, 10 μm). Control group: *n* = 5; TMZ group: *n* = 4; Fasudil group: *n* = 3; TMZ+Fasudil group: *n* = 7. Statistical differences compared with the control group is given as *P < 0.05, **P < 0.01, compared with the TMZ group is given as ^#^*P *< 0.05, ^##^*P *< 0.01, and compared with the fasudil group is given as ^&^*P* < 0.05, ^&&^*P* < 0.01

C6R cells (Supplementary Figure S6) were injected into the cerebral cortex of rats to establish an orthotropic transplantation TMZ resistance glioma model. Survival analysis revealed that the median survival of TMZ, control and fasudil group was 16, 15 and 16 days, respectively, whereas the median survival of the TMZ plus fasudil group was more than 25 days (Fig. 6a). The average body weight of the TMZ plus fasudil group was higher than that of the other groups (Fig. 6b). At day 25, MRI analysis showed that the tumor volume in the combination group was remarkably reduced compared with the other groups (Fig. 6c). Rats were killed and the brain sections were analyzed at day 25. The tumor size of the combination group was significantly smaller than that of the other groups (Fig. 6c). Immunohistochemical (IHC) results showed that p-ROCK2, p-moesin, p-β-catenin and ABCG2 were reduced in the group of TMZ+fasudil (Fig. 6d). Taken together, our data demonstrated that fasudil combined with TMZ inhibited the growth of TMZ-resistant gliomas in vivo.

### Fasudil increased sensibility of TMZ to suppress GSC growth via ROCK2 inhibition

**Fig. 7:**
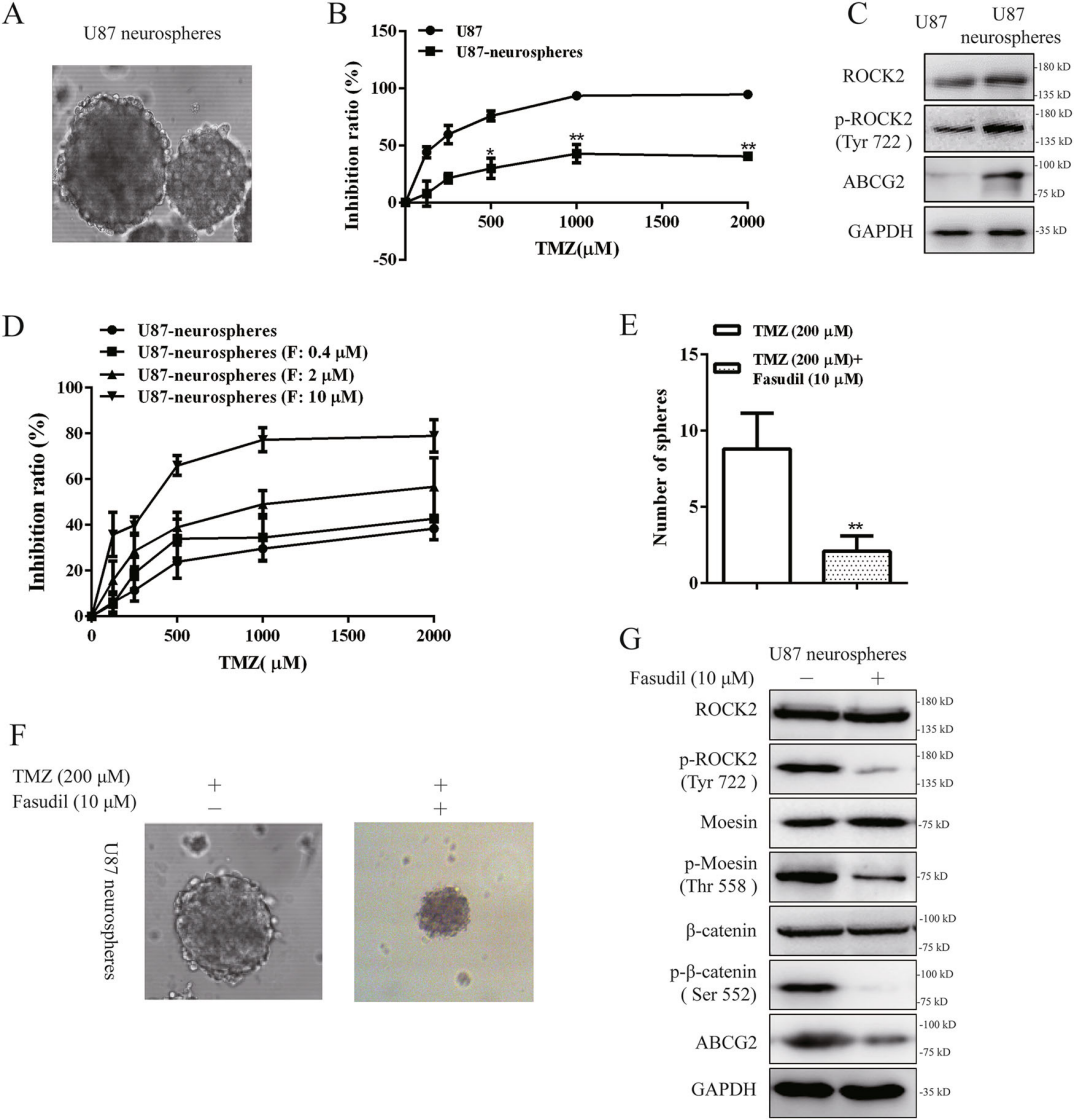
Fasudil increased sensibility of TMZ to suppress GSCs growth via ROCK2 inhibition. **a** The morphology of tumor spheres formed by U87 cell in SFM (×200). **b** Cells were treated with various concentrations of TMZ for 48 h, and cell viability was determined by the MTT assay.** c** Protein expression of ROCK2, p-ROCK2 (Tyr 772) and ABCG2 was detected in U87 and U87 neurospheres cells. **d** U87 and U87 neurospheres cells were treated with different concentrations of fasudil (0.4, 2, 10 μM) and TMZ (0, 125, 250, 500, 1000 and 2000 μM) and then cell viability was determined by the MTT assay. **e** U87 neurosphers were treated with TMZ (200 μM) plus fasudil (10 μM), and numbers of sphere were counted. **f** The morphology of tumor spheres is shown with stimulation of TMZ (200 μM) plus fasudil (10 μM) (×200). **g** ROCK2, p-ROCK2 (Tyr 722), moesin, p-moesin (Thr 558), β-catenin, p-β-catenin (Ser 552) and ABCG2 expression was determined by western blot with fasudil (10 μM). GAPDH was used as a loading control. Statistical differences compared with the control group is given as *P < 0.05, **P < 0.01

Recent reports suggested that the glioma stem cell (GSC) population serve as a source of chemo- and radiation-therapy resistance^38^. We cultured the U87 cells in serum-free medium (SFM) and observed the formation of tumor spheres. U87 cells could form floating neurospheres in less than a week (Fig. 7a). The flow cytometry analysis indicated that the percentage of CD133+/CD44+ cells in U87 tumor spheres was higher than U87 cells (Supplementary Figure S7A and S7B). In addition, IF staining showed that the expression of nestin was determined (Supplementary Figure S7C). In addition, U87 neurospheres were resistant to TMZ by MTT (3-(4,5-dimethylthiazol-2-yl)-2,5-diphenyltetrazolium bromide) assay (Fig. 7b). We found that expression of ROCK2, p-ROCK2 and ABCG2 proteins was higher in sphere cells than monolayer cells (Fig. 7c). With stimulation of fasudil, U87 neurospheres exhibited upregulation of sensibility to TMZ (Fig. 7d, Table 1). To investigate the inhibitory effect on growth of GSCs, GSCs were treated with TMZ (200 μM) plus fasudil (10 μM). In 5-day neurosphere formation assays, the size and number of neurospheres were reduced with inhibition of p-moesin, p-β-catenin and ABCG2 expression (Fig. 7e–g). Collectively, our data demonstrated that fasudil combined with TMZ inhibited the growth of GSCs.

## Discussion

**Fig. 8:**
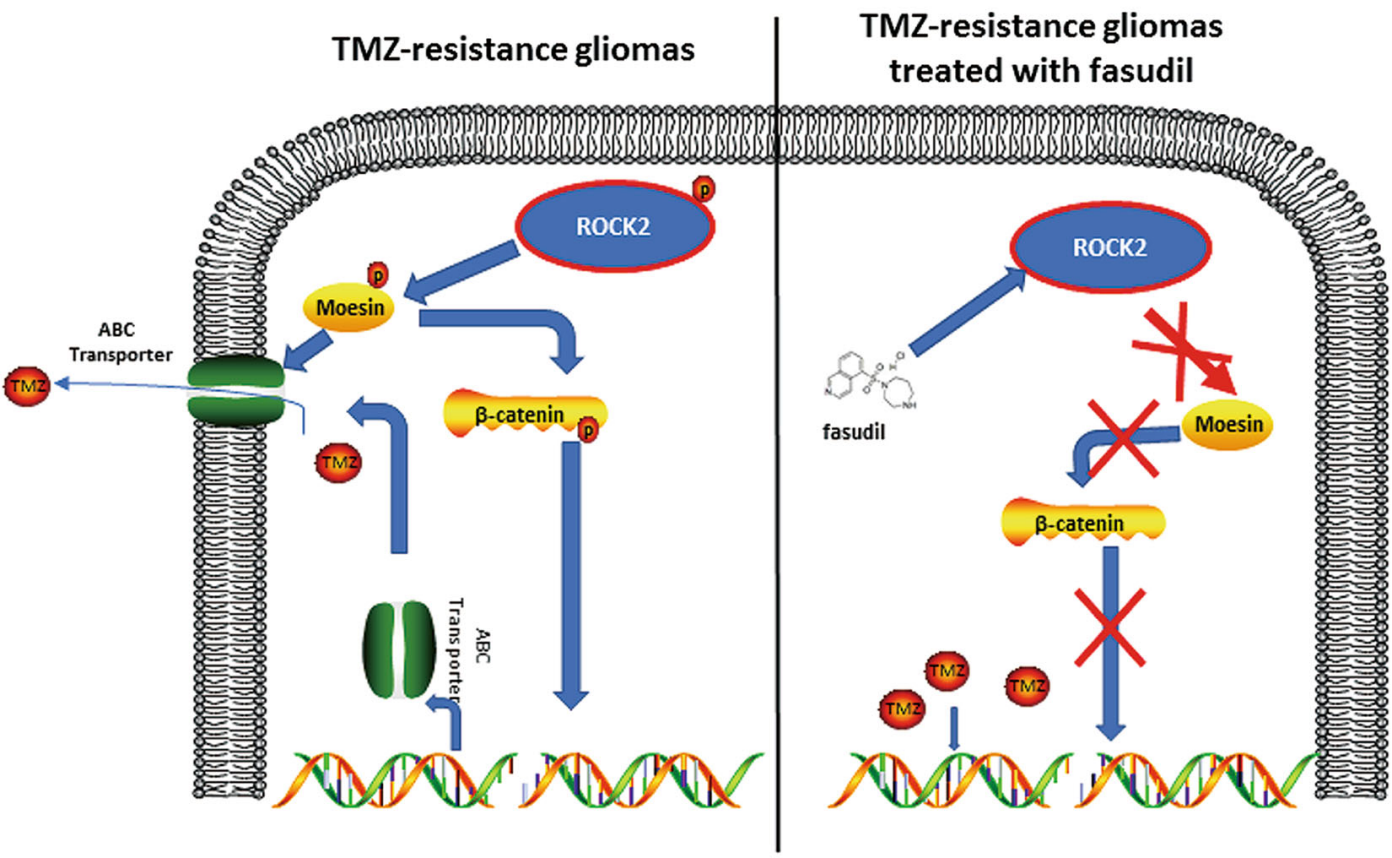
The pathway involved in TMZ-R gliomas with fasudil treatment

TMZ is a component of standard post-resection chemotherapy for glioma treatment^2^. However, mortality rate often does not decline after TMZ treatment due to therapeutic resistance and tumor recurrence^39^. As a key regulator of the cytoskeleton, the role of ROCK in chemoresistance has been widely reported^17,22^, although the underlying mechanism remains unclear. In our study, we found that fasudil increased sensitivity of TMZ in resistance cells via the ROCK2/moesin/ABCG2 pathway (Fig. 8). The combination of TMZ and fasudil reversed the resistance of TMZ in our TMZ-R models.

ROCK1 inhibition resulted in death of cancer cells^11^. In addition, ROCK1 is a regulator of apoptosis and autophagy^16^. ROCK2 is located at cytoplasm and can be recruited to cell membranes after being activated, playing an important role in regulating actin stress^16^. ROCK1 has been detected in non-neuronal tissue, and ROCK2 was strongly expressed in the central nervous system^16^. We have demonstrated that phosphorylation of ROCK2 instead of ROCK1 was increased in our TMZ-R models of glioma. We speculate that ROCK1 and ROCK2 have distinct functions in different types of resistance tumors.

In this study, we demonstrated that fasudil plus TMZ could be an effective combination therapeutic strategy for TMZ-R gliomas in pre-clinical research. Numerous ROCK inhibitors have been developed over the past two decades. However, only two inhibitors, fasudil and ripasudil, have been applied to clinical use^11^. The application of ROCK inhibitors for clinical use was limited due to side effects such as conjunctival hyperemia, effects on blood pressure and a narrow therapeutic window^11^. The side effects of ROCK inhibitors resulted in the discontinuation of some pre-clinical and clinical candidates, including Y-39983^40,41^, AR-12286^42,43^ and DE-104^44^. Although ROCK inhibitors have been well described for the treatment of cancer in literature, the potential systemic side effects have limited their application in cancer therapy. Numerous studies are required to further understand the role of ROCKs in oncology. Our study may suggest a new potential application for ROCK inhibitors for treatment of TMZ resistance in gliomas.

The ROCK/ERM/ABC-transporter pathway was found to be important in the small intestinal cell membrane for drug distribution^24,25^. Here, we reported that this pathway participated in TMZ-R glioma, and it could be disrupted by fasudil, leading to the dislocation of ABCG2 via suppression of p-moesin. Our finding suggested that p-moesin was regulated by ROCK2 in TMZ-R cells. The activation of moesin requires two steps. First, the structure of the inactive form (head to tail between intramolecular or intermolecular) must be broken by the transmembrane protein to expose its phosphorylation sites, and then the phosphorylation sites can be phosphorylated by kinases^45^. In our study, we found that the phosphorylation of moesin was regulated by ROCK2, but the mechanism of the disruption for the inactive form remains unknown. We also found that expression of ABCG2 was regulated by nuclear translocation of β-catenin. Our study agrees with previous studies showing that elevated phosphorylation of moesin increased β-catenin phosphorylation (Ser 552) and induced the nuclear translocation of β-catenin, which regulates the expression of ABCG2^46,47^. Although ABC drug efflux transporters in tumor cells confer multidrug resistance on several solid tumors, their roles in gliomas need more researches to reveal their function and regulation. Our data revealed a novel mechanism for ABCG2 regulation in TMZ-R gliomas. Compared with normal brain tissues, ABCG2 expression in GBM vessels and parenchymal tissue exhibited a higher level^48^. Moreover, high-grade gliomas tended to have an enhanced expression of ABCG2 compared with low-grade gliomas^49^. In our study, we also found that ABCG2 was overexpressed in resistance cells, indicating that ABCG2 may play important roles in TMZ-R gliomas.

ABC transporters were important for BBB^50^. Inhibition of ABC transporters may open BBB for drug distribution^51^. The results of MRI showed that BBB could alter in patients of GBM; however, the alteration of BBB may be negligible for glioma and accumulation of chemotherapeutic drugs^52,53^. In our study, expression of ABCG2 was downregulated with fasudil. Hence, we speculated that fasudil may open BBB and increase accumulation of TMZ for brain tumor treatment. Next, the in situ glioma animal models are needed to warrant this speculation for further investigation.

Substrates for ROCKs include the myosin-binding subunit of myosin phosphatase (MYPT1) and MLC, which regulate actomyosin contractile forces^54–56^. Recent researches lacked evidence for actin cytoskeleton function in cancer chemoresistance. Remarkedly, we found that cells displayed a ‘tail retraction’ phenotype with fasudil for more than 3 days, a well-known characteristic of ROCK inhibition^57^. This phenotype meant that cells lacked stress fibers. Thus, we speculated that stress fibers may be involved in TMZ-R, and this will open a new explore avenue.

As mentioned above, the drug resistance in cancer cells is often associated with stem cell components. It was reported that overexpression of CD133 promoted drug resistance^58^. In this study, we found that GSC proliferation was suppressed with fasudil plus TMZ, indicating that ROCK2 may play an important part in GSCs. Confusingly, it was reported that fasudil enhanced the cells’ ability to form spheres, and increased stem cell marker expressing GSC/BTIC-like cell subpopulation^59^. Nevertheless, studies revealed that ROCK inhibition led to growth suppression of CSCs. ROCKs were reported to regulate sensibility of gemcitabine in pancreatic cancer stem cells, and fasudil sensitized them to gemcitabine^20^. Rho/ROCK signaling is important for cancer stem cells (CSCs) to resist radiation therapy^15^. However, the role of ROCKs in CSCs remains unclear. Here, we reported that with abrogation of ROCK2 function, p-moesin, p-β-catenin and ABCG2 protein levels were decreased, and these results in our study preliminarily revealed a novel mechanism for maintaining resistance in CSCs.

In summary, our study revealed that fasudil increased the sensibility of TMZ and reversed TMZ resistance in glioma via the inhibition of ROCK2. This study added ROCK2 on the map of relevant molecular changes in TMZ resistance glioma and provided a therapeutic strategy for overcoming TMZ resistance in glioma therapy.

## Materials and methods

### Cell culture

Human U87, U251 and rat C6 glioma cell lines were obtained from the Type Culture Collection of the Chinese Academy of Sciences (Shanghai, China). U251 and U87 cells were authenticated with methods of short tandem repeat. Human glioma primary cells T5, T6 and rG-1 were gifts from Qing Wang (Department of Neurosurgery, Wuxi Second Hospital Affiliated Nanjing Medical University). All glioma cell lines and resistance cell lines were cultured in Dulbecco's modified Eagle's medium (DMEM; Keygen, Nanjing, China) containing 10% fetal bovine serum, and maintained in a humidified atmosphere of 95% air, 5% CO_2_ at 37 °C. The details of resistance cell lines can be found in the Supplementary Data section.

### Reagents and plasmids

TMZ was obtained from Changzhou new area Jili chemical, China. XAV939 and fasudil were from Selleck Chemicals Inc. (Houston, USA). LPA, Ko-143 and DOX were obtained from Sigma Life Science (MO, USA). For in vitro study, XAV939, Ko-143, DOX and TMZ were dissolved in 100% dimethyl sulfoxide (DMSO) with a concentration of 200 mM and stored at −20 °C. Fasudil and LPA were dissolved in phosphate buffered solution (PBS, pH = 7.4) with a concentration of 100 mM and stored at −20 °C. Samples containing 98% or higher were used in all experiments unless otherwise indicated. The working solution was freshly prepared in the basal medium. Controls were always treated with the same amount of DMSO as used in the corresponding experiments. For in vivo study, TMZ and fasudil were dissolved in PBS (pH = 4) and used immediately after they were ready. Sources of the antibodies were as follows: anti-ezrin, anti-radixin, anti-MLC2, anti-p-MLC2, anti-moesin and anti-p-β-catenin (Ser 552) were obtained from CST Technology Inc. (MA, USA). Anti-β-catenin, anti-p-ezrin (Thr 567), anti-ROCK2, anti-p-ROCK2 (Tyr 722) were purchased from Abcam (MA, USA). Anti-Lamin A and anti-ABCG2 were acquired from Bioworld Technology Inc. (MN, USA). Anti-p-moesin (Thr 558) was from Santa Cruz Biotechnology (CA, US). Anti-GAPDH was from Proteintech Group (IL, USA). The pSIN4-EF2-ABCG2-IRES-Neo was a gift from Ren-he Xu (Addgene plasmid # 25983)^60^. The control plasmids and anti-p-radixin (Thr 564) were gifts from Dr. Haiwei Zhang, Chongqing Cancer Hospital. The small interfering RNAs (siRNAs) of moesin, β-catenin and control were obtained from Santa Cruz Biotechnology (Santa Cruz Biotechnology Inc., CA). The sequence of siRNA-ABCG2 was 5’-CTGGAGATGTTCTGATAAA-3’, and sequence of siRNA-ROCK2 was 5’-GCAGACAAGAAACGAAAUUUG-3’. siRNAs of ROCK2 and ABCG2 were synthesized by Shanghai Sangon (Shanghai, China).

### Western blotting analysis

For total cell lysis, cells were lysed in extraction buffer (Beyotime, Hangzhou, China) for 1 h on ice. The lysates were centrifuged at 12,000×*g* for 20 min. For extracting the membrane protein, a membrane and cytosol protein extraction kit (Beyotime, Hangzhou, China) was used following the instructions of the manufacturer. The protein concentration was quantified by bicinchonininc acid (BCA) assay. Western blots were performed as previously described^61^.

### Real-time PCR analysis

Total RNA was extracted using TRIzol Reagent (Invitrogen). Total RNA was reverse-transcribed to complementary DNA (cDNA) using first-strand cDNA synthesis superMix kit (TransGen Biotech, Beijing, China). Real-time PCR was performed using the AceQ qPCR SYBR Green Master Mix Kit (Vazyme Biotech, Nanjing, China). The primer sequences used in this study are shown in supplementary tables S2 and S3.

### GSC neurosphere formation assays

The SFM was used to culture stem-like glioma cells. SFM was composed of DMEM/F12 (Gibco, Grand Island, NY), 20 ng/ml basic-fibroblast growth factor (Peprotech Inc., NJ, US), 20 ng/ml epidermal growth factor (Peprotech Inc.), 2% B27 supplement (Gibco), 1% glutamine (Sigma Life Science, MO, USA) and 1% non-essential amino acids (Gibco). U87 cell was dissociated and washed with PBS 3 times, and 10^6^ single cells were seeded in triplicate in 60 mm low-binding dishes (Becton Dickinson, San Jose, US) containing 6 ml SFM.

### Flow cytometry

Cells were starved in serum-free medium for 6 h to be synchronized, and after treatment with fasudil (0.4, 2, 10 μM) for 24 h, the medium was changed and cells were treated with DOX (20 μM) for 1 h. Finally, cells were analyzed by Accuri C6 Software flow cytometry (Becton Dickinson, San Jose, USA).

### RhoA GTP pull-down assay

For RhoA activation assay, a RhoA pull-down activation assay biochem kit (bead pull-down format, BK-036, Cytoskeleton, Denver, USA) was used following the instructions of the manufacturer. RhoA (Total), RhoA GTP and RhoA GDP expressions were determined by western blots.

### Immunoprecipitation assay

Aliquots of cell lysates were incubated with antibodies at 4 °C for 2 h and then precleared with protein-A/D-Sepharose (Beyotime, Hangzhou, China) at 4 °C overnight. Immunoprecipitated complexes were subjected to western blot with the primary antibody, followed by peroxidase-conjugated appropriate secondary antibody and visualized by 5200 chemiluminescence imaging system (Tenon, Shanghai, China).

### Knockdown and overexpression assay

Cells were transfected with siRNA, control-siRNA, plasmid and control plasmid with ExFect Transfection Reagent (Vazyme Biotech, Nanjing, China) for 24 h.

### In vivo study

U251R cells (1.0 × 10^6^ cells per mouse) were subcutaneously injected into 40 nude mice (Model Animal Research Center of Nanjing University, MARC). When the tumor volume reached 50 mm^3^, mice were randomly divided into 4 groups (10 mice per group). Mice were treated with TMZ (25 mg/kg), fasudil (20 mg/kg), TMZ (25 mg/kg) plus fasudil (20 mg/kg) and solvent through caudal veins. The treatment was performed once every 2 days for 21 days. The tumor volume was measured with a caliper every 2 day, using the following formula: volume = length × width^2^/2.

Sprague–Dawley rats (obtained from MARC) were anesthetized with 3% pentobarbital sodium, and a 1.0-mm-diameter hole was drilled through the skull. C6R cells (1.0×10^6^ cells per rats) in 10 μl PBS were injected intracerebrally into the striatum. After surgery, rats were randomly divided into 4 groups (12 rats per group), and treated with TMZ (35 mg/kg), fasudil (30 mg/kg), TMZ (35 mg/kg) plus fasudil (30 mg/kg) or solvent alone through abdominal cavity injection once every 2 days. The treatment was performed once every 2 days for 25 days.

All procedures were approved and performed in accordance with the guidelines of the ethics approval from the Experimentation Ethics Review Committee of China Pharmaceutical University.

### Immunohistochemistry

Tissues were sent to make paraffin section by the Nanjing Drug Toxicology Research Institute. Blocking was performed by incubating 2 h at room temperature with PBS containing 10% goat serum. Then, sections were incubated with primary antibodies for 2 h at 4 × degrees celsius. Then the immunohistochemistry kit of Maixin Biotech was used. The immunoreactivity was finally examined by NanoZoomer 2.0 RS (Hamamatsu Photonics Co., Ltd, Shizuoka, Japan).

### Immunofluorescent staining

Cells were washed with PBS and fixed with methyl alcohol for 20 min at −20 °C, and then cells were blocked with PBS containing 3% bovine serum albumin for 1 h at 37 °C. After being incubated with the primary antibody overnight at 4 °C, cells were treated with the secondary antibody (Keygen, Nanjing, Chian) for 1 h at 37 °C. Nuclei were stained with 4’,6-diamidino-2-phenylindole (obtained from Santa Cruz Biotechnology) and IF photomicrographs were captured using a fluorescent microscope (Carl Zeiss, Germany).

### Statistical analysis

All experiments reported here were performed with triplicate independent replications. Results are presented as the mean ± S.D. Significance between two groups was assessed by Student’s two-tailed* t*-test. The non-parametric Mann–Whitney *U*-test was used to assess significance between two means of data sets lacking a normal distribution and having a small sample size. Data sets consisting of more than two groups were analyzed by analysis of variance with Tukey–Kramer honest significant difference post test for multiple comparisons if significance was determined. *P*-value that was less than 0.05 was considered statistically significant for all data sets. All statistical analyses were performed using GraphPad Prism software.

## Electronic supplementary material

Supplementary Information

Supplementary Figure

Supplementary Figure

Supplementary Figure

Supplementary Figure

Supplementary Figure

Supplementary Figure

Supplementary Figure

Supplementary TableS1

Supplementary TableS2

Supplementary TableS3
