# Supplementary material for: Fasudil increases temozolomide sensitivity and suppresses temozolomide-resistant glioma growth via inhibiting ROCK2/ABCG2
Source: Cell Death Dis. 2018 Feb 7;9(2):190. doi: 10.1038/s41419-017-0251-9 (PMC5833824; doi:10.1038/s41419-017-0251-9)
Supplement: Supplementary file 1 — Supplementary Information [file 41419_2017_251_MOESM1_ESM.docx]

**Supplementary files**

**Materials and methods**

**Generation of TMZ-resistant cell lines**

All glioma cells were exposed to increased concentrations starting at 50 μM of TMZ and doubling the concentration every 2 weeks until cells became resistant to 1000 μM. Cells were routinely tested for TMZ resistance. All resistance and parental cells were plated into 96-well plates (Corning, New York, NY) at 3,000 cells/well, and then treated with various concentrations of TMZ (0, 125, 250, 500, 1000, or 2000 μM) for 48 h. Cell viability was assessed with the MTT assay. The degree of resistance was estimated by resistant index (RI). Cells that acquired resistance to TMZ were labeled as C6R, U87R, U251R, T5R, and T6R. All assays were performed in triplicate.

**Colony-formation assays**

Resistance and parental cells were seeded in 12-well plates at a density of at 100 cells per well. Cells were treated with different concentrations of TMZ (0, 12.5, 25, 50 and 100 μM) for 7 days. Plates were further cultured in an incubator until colonies were large enough to be visualized. Colonies were counted with ten fields in one well under a TS-100 inverted microscope (Nikon Co., Ltd, Yokohama, Japan).

**Flow cytometry analysis**

Cells were washed twice with PBS and collected, then stained for fluorescence-activated cell sorting (FACS) analysis. For cell surface antigen staining, cells were incubated with the appropriate concentration of FITC-conjugated anti-CD44 and PE-conjugated anti-CD133 antibody (Ab) for 30 min on ice. After washing with phosphatebuffered saline, cells were acquired by a FACSCalibur using Cell Quest software (both from BD Biosciences) and were analyzed by FlowJo software (Tree Star, Inc, Ashland, OR).

**Figure Legends**

Figure S1. Determination of TMZ-resistant cell line. (A) Cells were treated with various concentrations of TMZ for 48 h, and then cell viability was determined by the MTT assay. (B) The colony formation ability of TMZ-resistant glioma cells or parental glioma cells with stimulation of TMZ (0, 12.5, 25, 50 and 100 μM μM) for 1 week. (C) mRNA expression of mgmt was detected in all cells. (D) MGMT protein level was determined in rG-1 and U251R cells. Data were expressed as means ± SD of three independent experiments. Statistical differences compared with the controls are given as *P < 0.05, **P < 0.01.

Figure S2. The RhoA was activated in TMZ-R cells. A: U251/U251R, B: U87/U87R, C: T5/T5R, D:T6/T6R, E:C6/C6R, F:rG-1.

Figure S3. The effect on proliferation with ROCK2 knock-down. (A) rG-1 cell was transfected with siRNA-ROCK2, after 3 days, cell was stained with crystal violet (Upper). Cell was transfected with siRNA-ROCK2, then TMZ (400 μM) was stimulated with cell for 3 days and cell was stained with crystal violet (Down). (B) U251R, U87R, T5R, T6R and rG-1 were knocked-down ROCK2, then cell viability was determined by the MTT assay under stimulation of TMZ (0, 125, 250, 500, 1000, or 2000 μM) for 48 h. Data were expressed as means ± SD of three independent experiments. Statistical differences compared with the controls are given as *P < 0.05, **P < 0.01.

Figure S4. The anti-proliferation effect of ABCG2 inhibition. (A) U251R cell was knocked-down ABCG2 or treated with Ko-143 (15 μM), cell viability was determined by the MTT assay with TMZ (0, 125, 250, 500, 1000, or 2000 μM). (B) rG-1 cell was knocked-down ABCG2 or treated with Ko-143 (15 μM), cell viability was determined by the MTT assay with TMZ (0, 125, 250, 500, 1000, or 2000 μM). Data were expressed as means ± SD of three independent experiments. Statistical differences compared with the controls are given as *P < 0.05, **P < 0.01.

Figure S5. The expression of ezrin and radixin in all cells. (A). Ezrin and p-ezrin expressions were determined. (B). Radixin and p-radixin levels were detected.

Figure S6. Determination of the C6R cell lines and detection of ROCK2 and ABCG2 protein levels. (A) Cells were treated with various concentrations of TMZ for 48 h, and then cell viability was determined by the MTT assay. (B) C6R were knocked-down ROCK2, then cell viability was determined by the MTT assay under stimulation of TMZ (0, 125, 250, 500, 1000, or 2000 μM) for 48 h. (C) Protein levels of ROCK2, p-ROCK2 and ABCG2 were detected by western blot. (D) The colony formation ability of C6R or C6 cells with stimulation of TMZ (0, 12.5, 25, 50 and 100 μM μM) for 1 week. (E) The gene expression of abcg2, abcg2, abcc1, p-gp, abcc6 and mrp2 were determined. Data were expressed as means ± SD of three independent experiments. Statistical differences compared with the controls are given as *P < 0.05, **P < 0.01.

Figure S7. Determination of U87-neurospheres. (A and B) CD44 and CD133 expression were detected by flow cytometry. (C) Nestin expression of U87-neurospheres was determined by IF (X200, Nestin: green).

**Tables**

Table S1. The IC50 of resistance cell lines and parental cell lines.

Table S2. The primers of realtime-PCR for human glioma cells.

Table S3. The primers of realtime-PCR for rat glioma cells.
