# Supplementary figures and images for: Fasudil increases temozolomide sensitivity and suppresses temozolomide-resistant glioma growth via inhibiting ROCK2/ABCG2
Source: Cell Death Dis. 2018 Feb 7;9(2):190. doi: 10.1038/s41419-017-0251-9 (PMC5833824; doi:10.1038/s41419-017-0251-9)

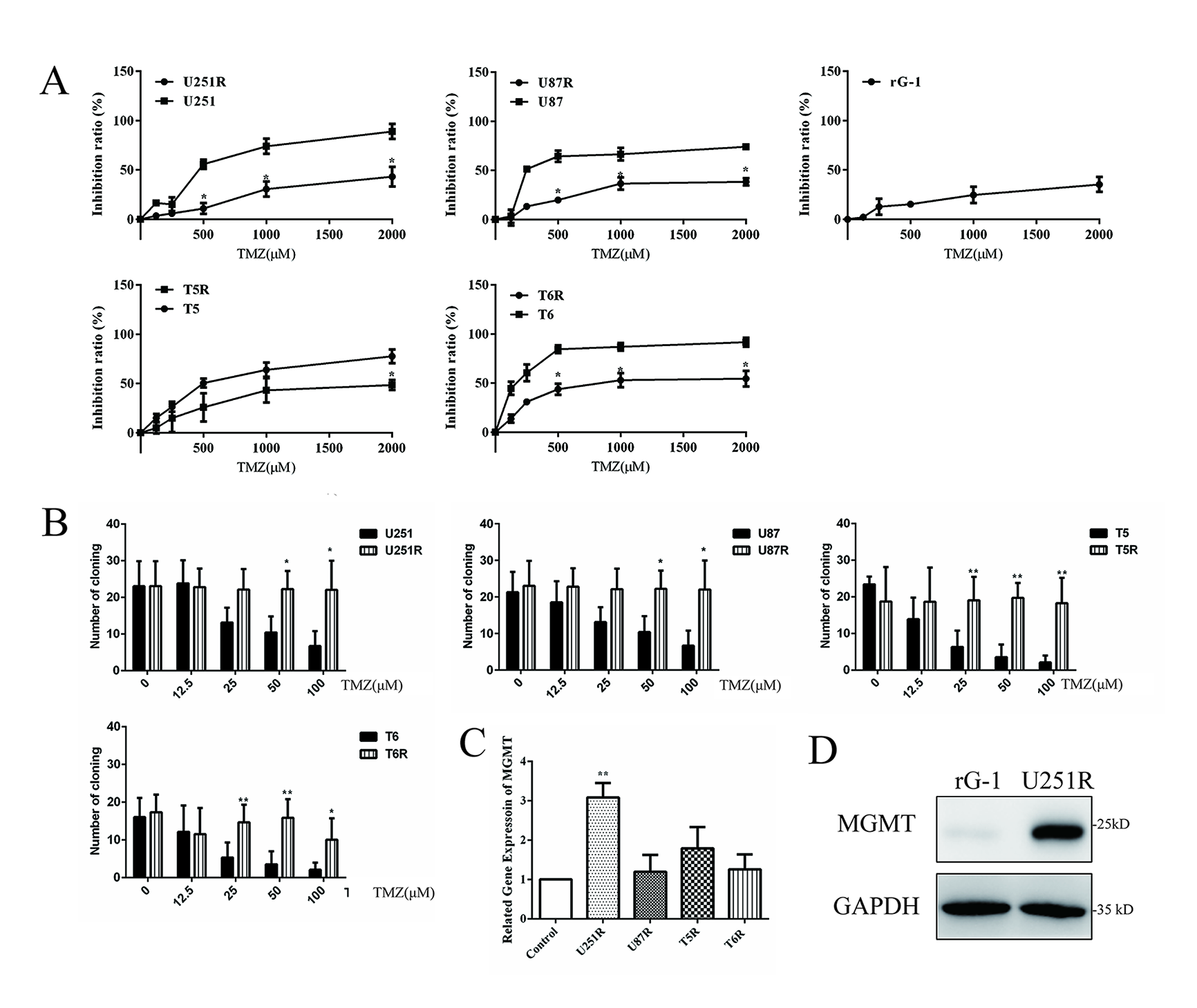

Supplement: Supplementary file 2 — Supplementary Figure [file 41419_2017_251_MOESM2_ESM.tif]

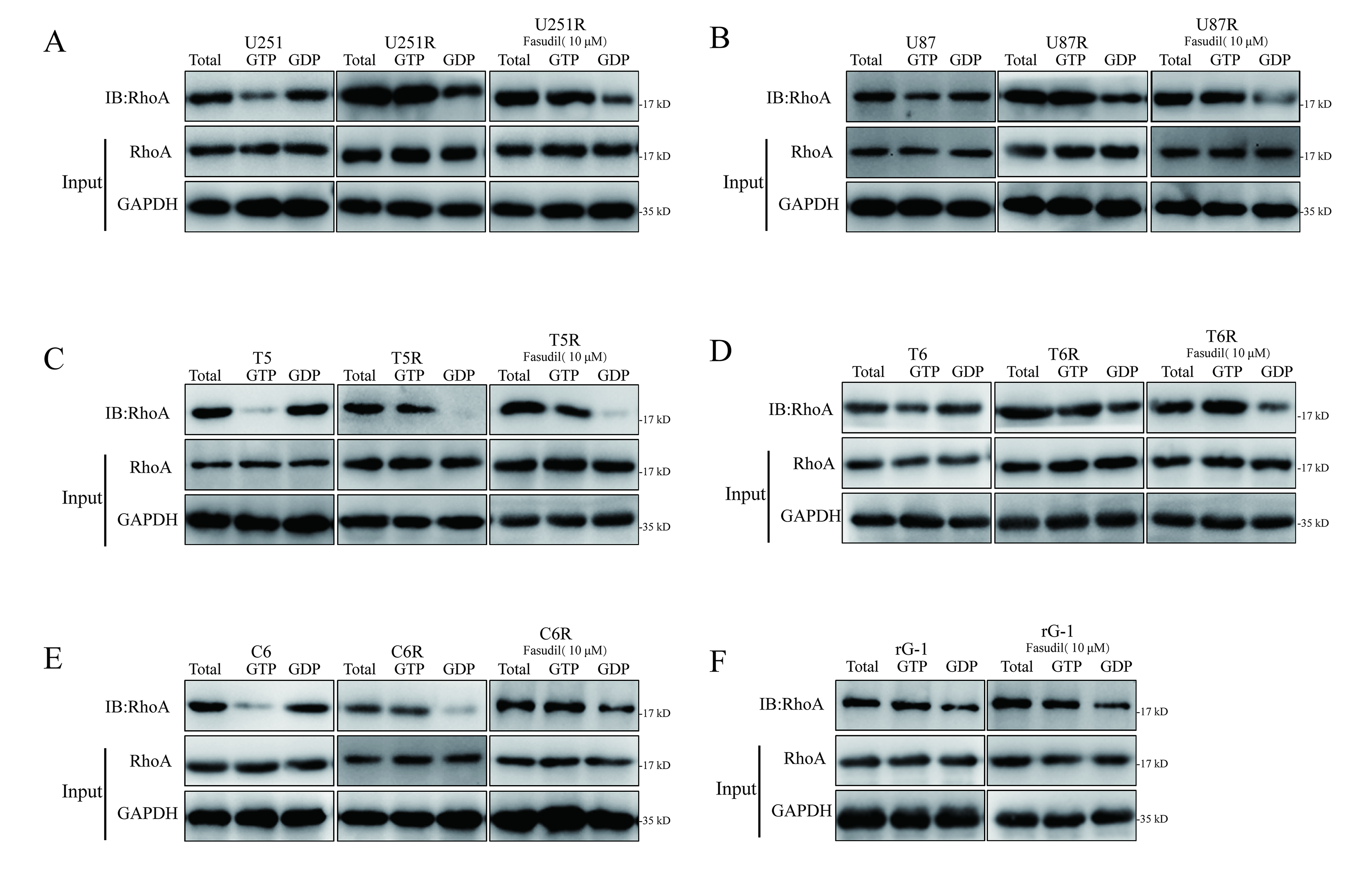

Supplement: Supplementary file 3 — Supplementary Figure [file 41419_2017_251_MOESM3_ESM.tif]

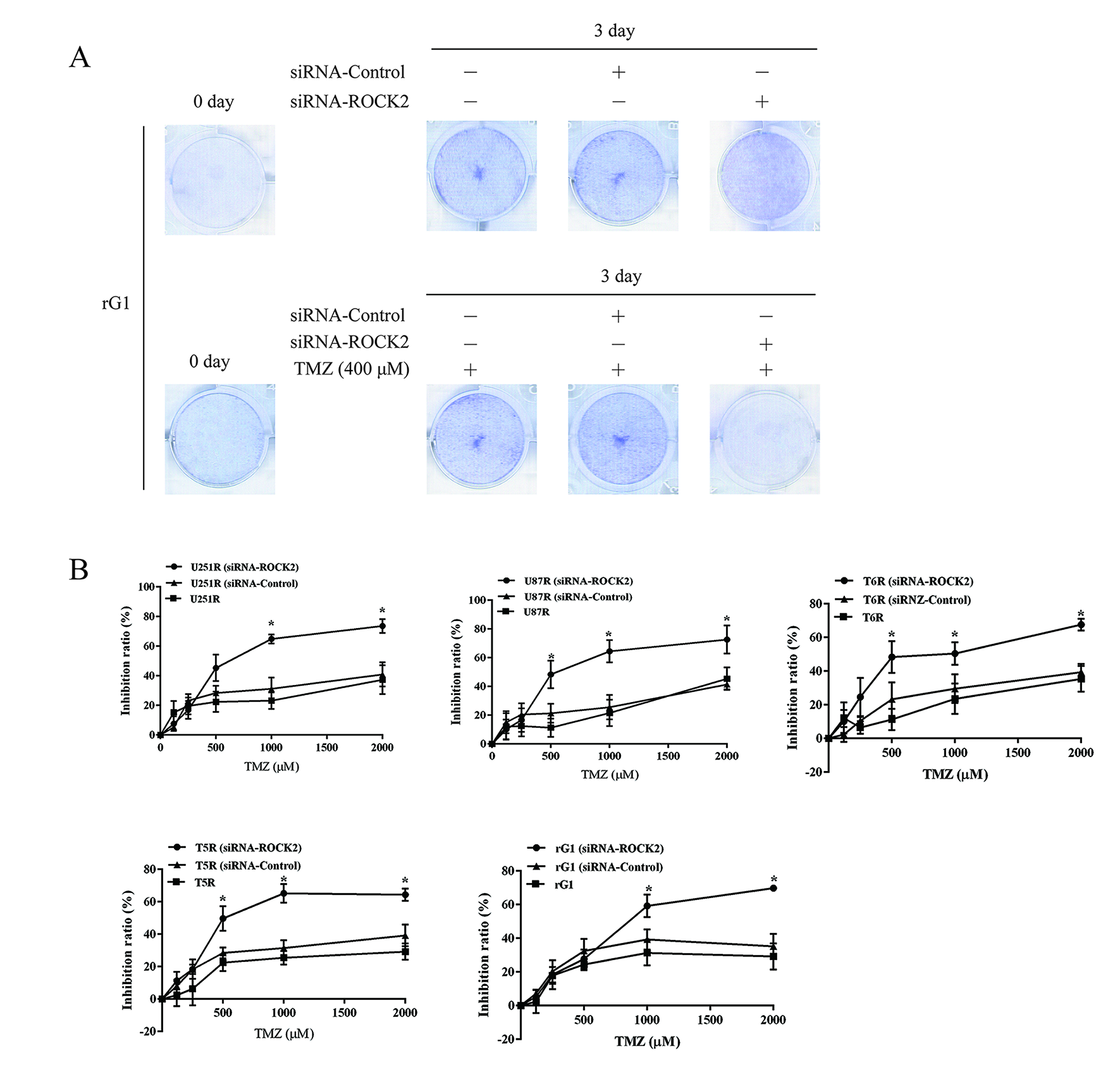

Supplement: Supplementary file 4 — Supplementary Figure [file 41419_2017_251_MOESM4_ESM.tif]

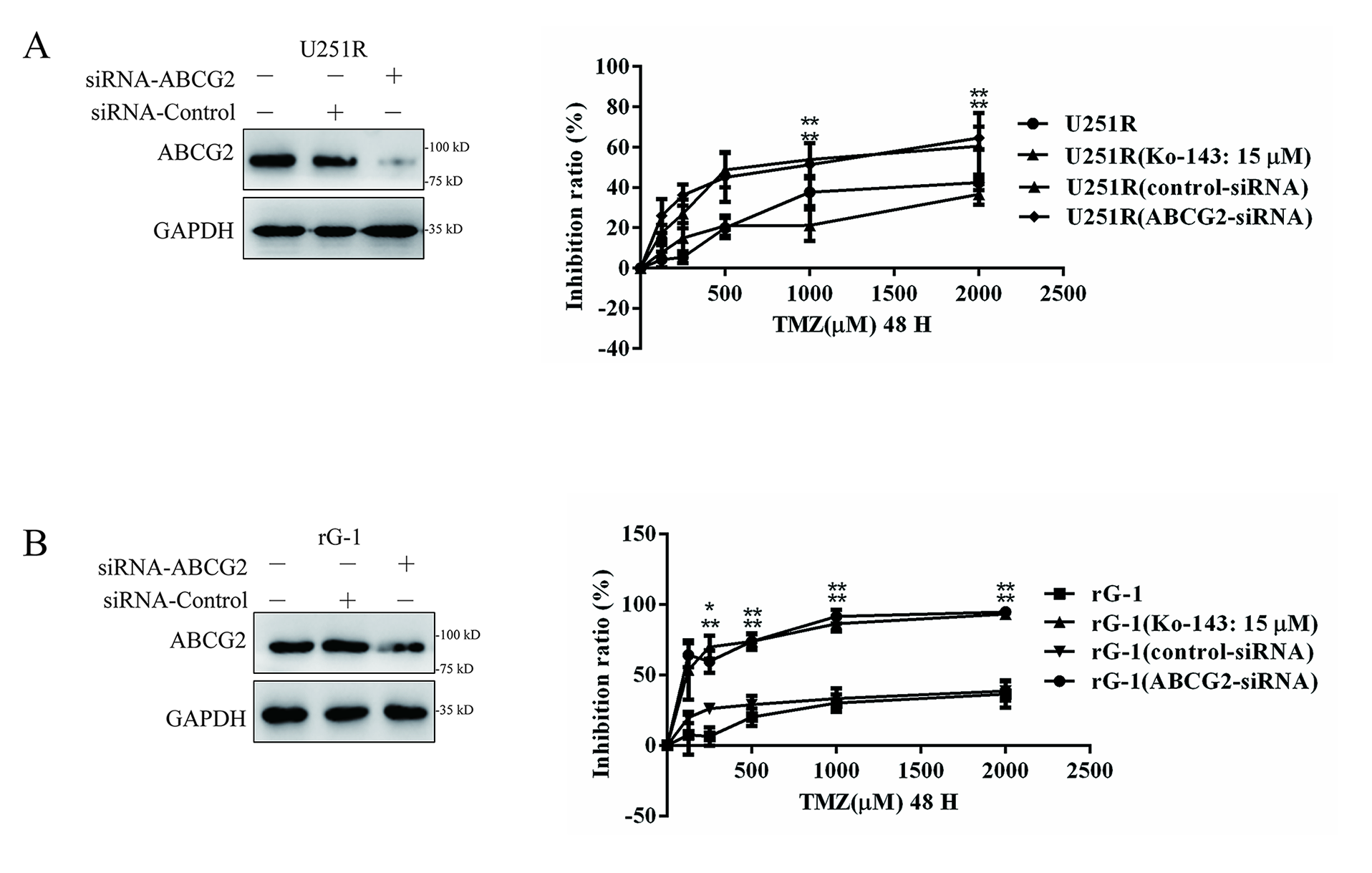

Supplement: Supplementary file 5 — Supplementary Figure [file 41419_2017_251_MOESM5_ESM.tif]

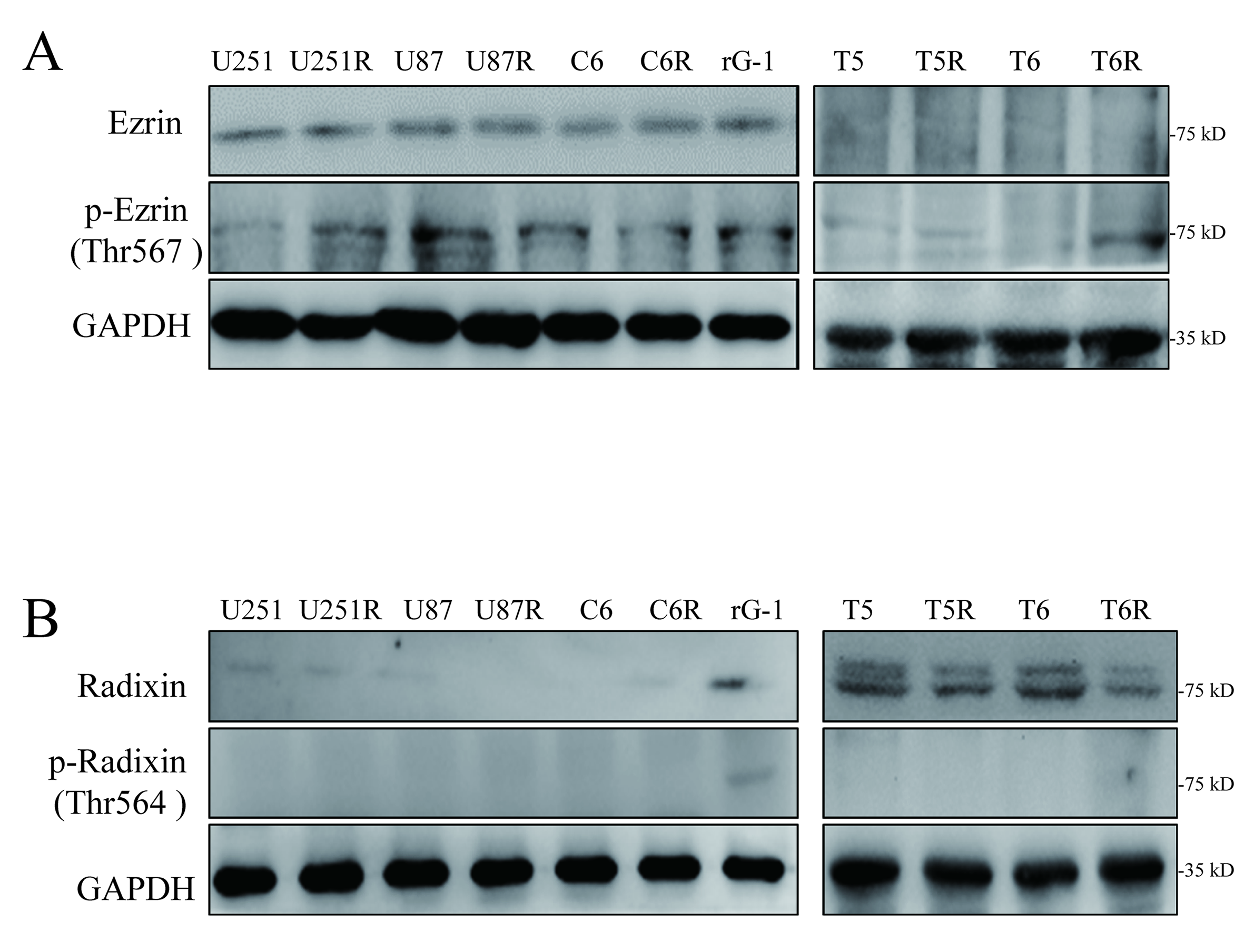

Supplement: Supplementary file 6 — Supplementary Figure [file 41419_2017_251_MOESM6_ESM.tif]

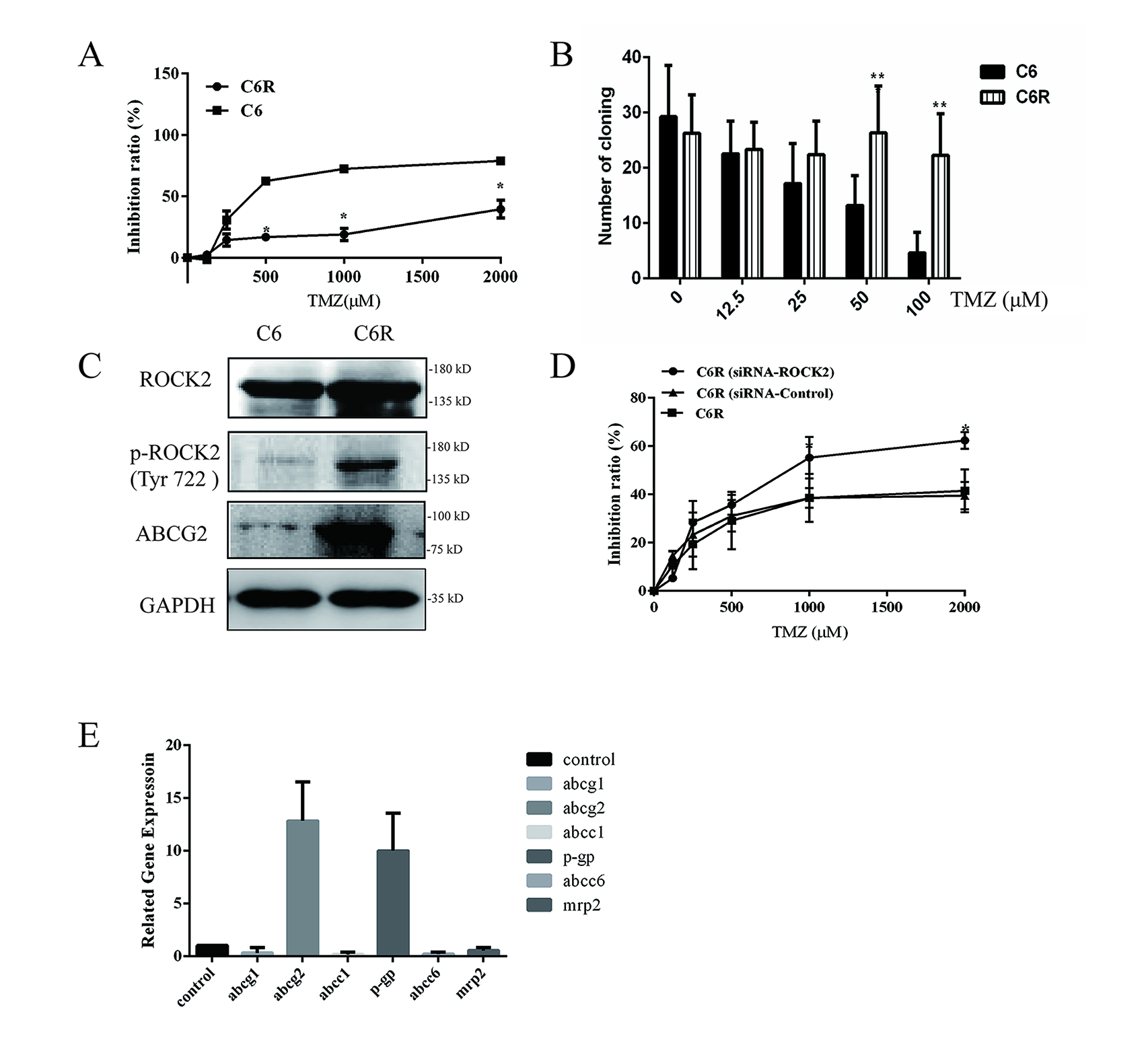

Supplement: Supplementary file 7 — Supplementary Figure [file 41419_2017_251_MOESM7_ESM.tif]

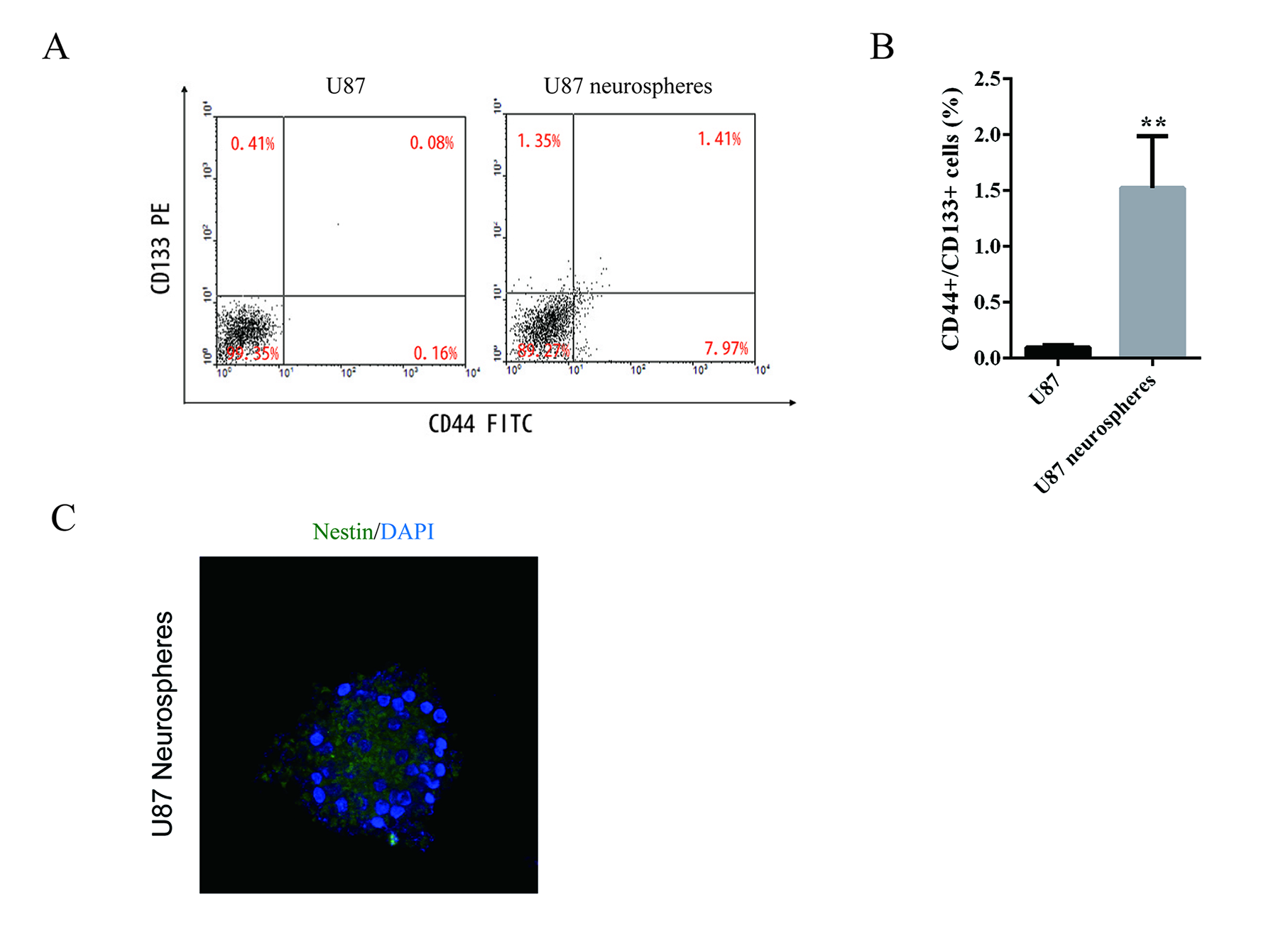

Supplement: Supplementary file 8 — Supplementary Figure [file 41419_2017_251_MOESM8_ESM.tif]
