# Supplementary material for: Fasudil increases temozolomide sensitivity and suppresses temozolomide-resistant glioma growth via inhibiting ROCK2/ABCG2
Source: Cell Death Dis. 2018 Feb 7;9(2):190. doi: 10.1038/s41419-017-0251-9 (PMC5833824; doi:10.1038/s41419-017-0251-9)
Supplement: Supplementary file 9 — Supplementary TableS1 [file 41419_2017_251_MOESM9_ESM.docx]

Table S1. The IC_50_ of resistance cell lines and parental cell lines.

| Cell lines | IC_50_ (μM) |
| --- | --- |
| U87 | 307.77±102.20 |
| U87R | 3437±770.61^**^ |
| U251 | 399.56±101.45 |
| U251R | 2832±388.63^**^ |
| C6 | 344.77±133.29 |
| C6R | 3280.66±415.31^**^ |
| T5 | 427.13±138.30 |
| T5R | 1953.33±161.91^*^ |
| T6 | 215.73±92.50 |
| T6R | 1521±492.59^**^ |
| rG-1 | 3437.33±638.39 |
