# Supplementary material for: Fasudil increases temozolomide sensitivity and suppresses temozolomide-resistant glioma growth via inhibiting ROCK2/ABCG2
Source: Cell Death Dis. 2018 Feb 7;9(2):190. doi: 10.1038/s41419-017-0251-9 (PMC5833824; doi:10.1038/s41419-017-0251-9)
Supplement: Supplementary file 10 — Supplementary TableS2 [file 41419_2017_251_MOESM10_ESM.docx]

Table S2. The primers of realtime-PCR for human glioma cells.

| Gene name | Forward primer | Reverse primer |
| --- | --- | --- |
| p-gp | 5’-GGTGCTGGTTGCTGCTTAC-3’ | 5’-AGCCTATCTCCTGTCGCATT-3’ |
| abcg1 | 5’-AAAGGGCTCGCTCAAGG-3’ | 5’-GGCAGTTCAGACCCAAATC-3’ |
| abcg2 | 5’-ATCCTTCCATCTTGTTCTTGG-3’ | 5’-CGTCCCTGCTTAGACATCCT-3’ |
| abcc1 | 5’-GAGGAAGGGAGTTCAGTCTT-3’ | 5’-ACAAGACGAGCTGAATGAGT-3’ |
| abcc6 | 5’-GCAACTGGACAGACCTAGAG-3’ | 5’-GAGCTCAGGTCGGTATCTTA-3’ |
| mrp2 | 5’-CACCATAAAGGACAACATCCTT-3’ | 5’-AGGCTGATCCGCTGCTTCTG-3’ |
| rock1 | 5’-GGGCGAAATGGTGTAGAAGA-3’ | 5’-AATCGGGTACAACTGGTGCT-3’ |
| rock2 | 5’-TGGATGAAACAGGCATGGTA-3’ | 5’-CATTCTCGCCCATAGAAACC-3’ |
| mgmt | 5’-CCTGGCTGAACCTATTTCC-3’ | 5’-GATGAGGATGGGGACAGGATT-3’ |
| gapdh | 5’-TGGTATCGTGGAAGGACTCA-3’ | 5’-CAGTAGAGGCAGGGATGATG-3’ |
