# Supplementary material for: Fasudil increases temozolomide sensitivity and suppresses temozolomide-resistant glioma growth via inhibiting ROCK2/ABCG2
Source: Cell Death Dis. 2018 Feb 7;9(2):190. doi: 10.1038/s41419-017-0251-9 (PMC5833824; doi:10.1038/s41419-017-0251-9)
Supplement: Supplementary file 11 — Supplementary TableS3 [file 41419_2017_251_MOESM11_ESM.docx]

Table S3. The primers of realtime-PCR for rat glioma cells.

| Gene name | Forward primer | Reverse primer |
| --- | --- | --- |
| p-gp | 5’- GGCCACATGATCAAGACG -3’ | 5’- AACAAGTTGCTGTTCTGCC -3’ |
| abcg1 | 5’- TCGTTCCTGCAACCTCGTC-3’ | 5’- TATAAAGGCGAATCGTGTCCC-3’ |
| abcg2 | 5’- CATGGTGGCATCCCTAGTAA -3’ | 5’- CGATTGCCTCACTCACTGTC -3’ |
| abcc1 | 5’- GTCAGCCCGACACTGCTAGGC-3’ | 5’- GTCTGAGAAGCAGGACAGCAC-3’ |
| abcc6 | 5’- CCTCTTCCTCAGCTTGGAGCT-3’ | 5’- GAAAGGAAATCCCGTAAATCC-3’ |
| mrp2 | 5’-AAGGTCGGGCATGGGCGGA-3’ | 5’- AGCTGTCGCTGCCCTACTCT-3’ |
| rock1 | 5’- TCCTTGGGTTGTTCAGCTTT-3’ | 5’- TTCAGGCACATCGTAGTTGC -3’ |
| rock2 | 5’- GGCCTTGCATATTGGTATGG -3’ | 5’- CAGCCATCCTTCTAATCGTGA -3’ |
| mgmt | 5’- GATGAGGAGCAATCCAGTCC -3’ | 5’-GCCAGAAGCCACTCTTTCAC -3’ |
| gapdh | 5’- TGGTATCGTGGAAGGACTCA -3’ | 5’- CAGTAGAGGCAGGGATGATG -3’ |
